# Expansion and functional analysis of the SR-related protein family across the domains of life

**DOI:** 10.1261/rna.079170.122

**Published:** 2022-10

**Authors:** Sean M. Cascarina, Eric D. Ross

**Affiliations:** Department of Biochemistry and Molecular Biology, Colorado State University, Fort Collins, Colorado 80523, USA

**Keywords:** SR protein, low-complexity domain, splicing, RNA-binding protein, RNA metabolism

## Abstract

Serine/arginine-rich (SR) proteins comprise a family of proteins that is predominantly found in eukaryotes and plays a prominent role in RNA splicing. A characteristic feature of SR proteins is the presence of an S/R-rich low-complexity domain (RS domain), often in conjunction with spatially distinct RNA recognition motifs (RRMs). To date, 52 human proteins have been classified as SR or SR-related proteins. Here, using an unbiased series of composition criteria together with enrichment for known RNA binding activity, we identified >100 putative SR-related proteins in the human proteome. This method recovers known SR and SR-related proteins with high sensitivity (∼94%), yet identifies a number of additional proteins with many of the hallmark features of true SR-related proteins. Newly identified SR-related proteins display slightly different amino acid compositions yet similar levels of post-translational modification, suggesting that these new SR-related candidates are regulated in vivo and functionally important. Furthermore, candidate SR-related proteins with known RNA-binding activity (but not currently recognized as SR-related proteins) are nevertheless strongly associated with a variety of functions related to mRNA splicing and nuclear speckles. Finally, we applied our SR search method to all available reference proteomes, and provide maps of RS domains and Pfam annotations for all putative SR-related proteins as a resource. Together, these results expand the set of SR-related proteins in humans, and identify the most common functions associated with SR-related proteins across all domains of life.

## INTRODUCTION

Low-complexity domains (LCDs) are regions in proteins with highly skewed amino acid compositions (Wootton 1994). While this simple defining feature distinguishes LCDs from non-LCD regions, LCDs can vary dramatically in their structures, functions, subcellular localization, and overall biophysical properties (Marcotte et al. 1999; Michelitsch and Weissman 2000; Sim and Creamer 2002; Albà and Guigó 2004; Faux et al. 2005; Harrison 2006; Simon and Hancock 2009; Radó-Trilla and Albà 2012; Lobanov et al. 2016; Chavali et al. 2017; Cascarina and Ross 2018; Cascarina et al. 2020, 2021), often depending on which amino acid(s) are predominantly enriched in each LCD sequence. Consequently, we and others have proposed additional layers of subclassification to adequately categorize LCDs (Harrison 2006; Radó-Trilla and Albà 2012; Cascarina et al. 2021).

One protein family prevalent in eukaryotic organisms, including humans, consists of SR proteins, which all contain an LCD enriched in serine and arginine (“RS domain”; Zahler et al. 1992; Long and Caceres 2009; Mueller and Hertel 2012). SR proteins play a quintessential role in messenger RNA (mRNA) splicing. The RS domains of SR proteins participate in a variety of functions including protein–protein interaction, protein–RNA interaction, nucleocytoplasmic transport, regulation by post-translational modification (PTM), and recruitment to and formation of nuclear speckles (Long and Caceres 2009; Mueller and Hertel 2012). RS domains can undergo phase separation (Tari et al. 2019) and mediate recruitment to membraneless organelles in a phosphorylation-regulated manner (Tari et al. 2019; Greig et al. 2020; Cascarina and Ross 2022). A recent mechanistic model of splicing proposed that these activities enable a complex splicing logic at the nuclear speckle interface, suggesting that the biophysical properties and behavior of RS domains are important features influencing splicing activity (Liao and Regev 2021). Additionally, although SR proteins are typically associated with splicing-related functions, these proteins participate in a wide variety of cellular processes, including nucleocytoplasmic shuttling, translation, chromatin organization, cell cycle regulation, and metabolism (Long and Caceres 2009; Shepard and Hertel 2009; Zhong et al. 2009; Giannakouros et al. 2011; Mueller and Hertel 2012; Wagner and Frye 2021; Slišković et al. 2022).

Classically, the canonical SR protein family consists of proteins with at least one amino-terminal RNA recognition motif (RRM) of sufficient homology with typical RRMs, a downstream RS domain at least 50 amino acids in length and >40% combined composition of R and S, and the presence of RS or SR dipeptide repeats within the RS domain (Zahler et al. 1992; Manley and Krainer 2010). In humans, this results in a well-defined family of 12 “SR splice factor” (SRSF) proteins. However, many more human proteins contain RS domains and are thus referred to as “SR-related proteins.” A number of these proteins also contain RNA-binding domains and are involved in mRNA splicing (Long and Caceres 2009) but do not adhere to the strict domain composition and organization defined by Manley and Krainer. Therefore, while such a narrow definition may be useful in defining the core SRSF protein family, the similarities both in terms of sequence features and biological functions warrant broader consideration and inclusion of SR-related proteins. For simplicity, we herein refer to SR proteins and SR-related proteins collectively as “SR/SR-related proteins” when the group contains both types of proteins.

Here, we use a composition-centric bioinformatic approach to identify 83 new SR-related proteins in humans—35 of which possess known RNA-binding activity—and test whether these candidates exhibit functional signatures consistent with previously identified SR/SR-related proteins. We find that the new candidate SR-related proteins resemble known SR/SR-related proteins in terms of domain composition, biological function, and post-translational regulation. RS domains from SR/SR-related proteins are substantially influenced by alternative splicing, often affecting inclusion or exclusion of exons containing the RS domain(s) in the final protein product, which in turn could affect the activities of SR/SR-related proteins in carrying out their splicing functions. Finally, we show that SR-related proteins are commonly associated with DEAD-box domains and/or helicases among archaea, bacteria, and eukaryotes, but often associated with protein self-assembly and viral nucleic acid packaging or processing in viruses, highlighting both commonalities and functional diversification of SR-related proteins across distinct domains of life.

## RESULTS

### Expansion of the human SR-related protein family

**FIGURE 1.**
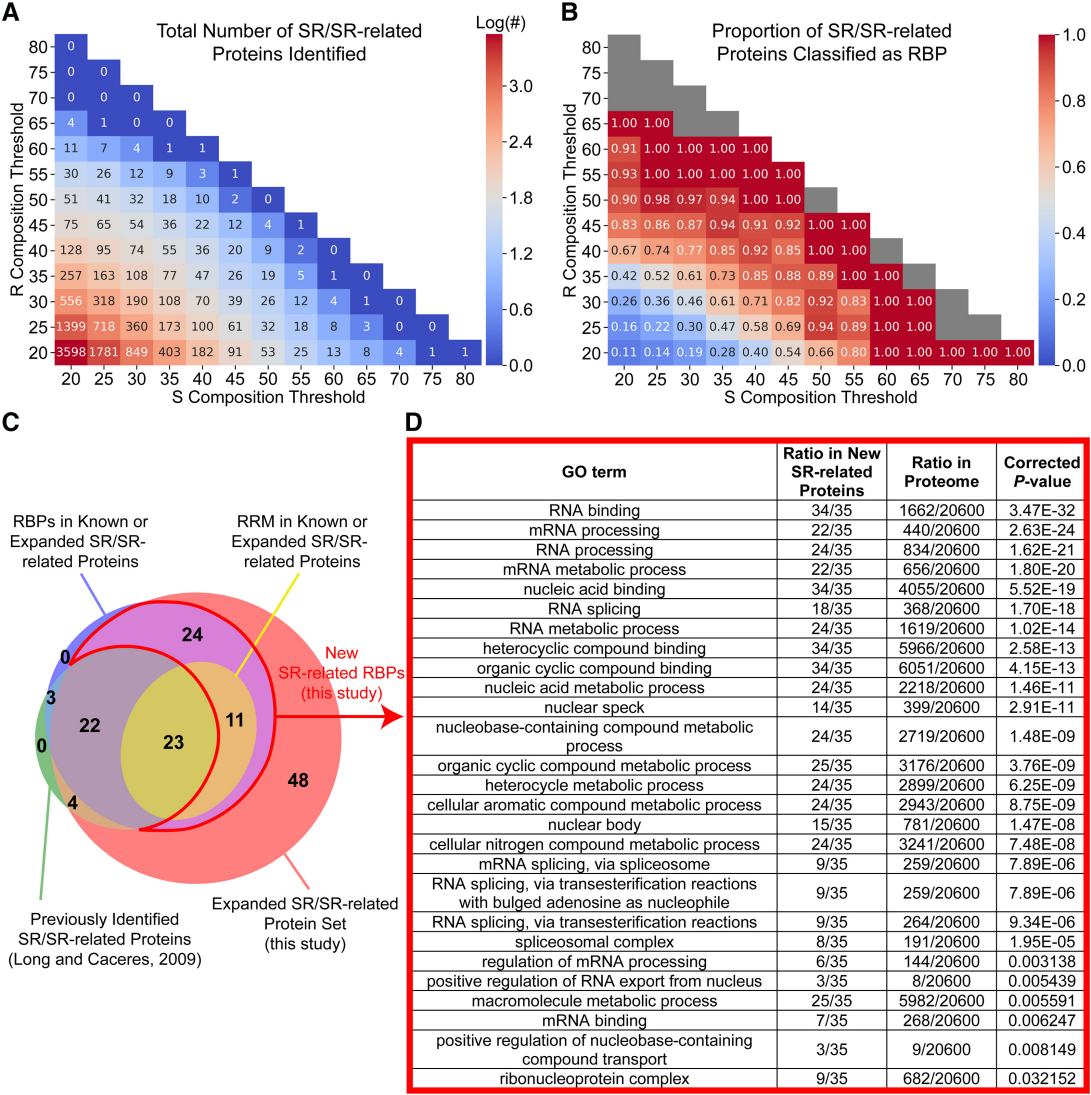
Identification of RS domains in human proteins. (*A*) The human proteome (UP000005640_9606; *n* = 20,600 proteins) was iteratively analyzed using LCD-Composer, each time using a unique combination of S composition threshold and R composition threshold. The heatmap indicates the number of proteins identified for each composition threshold criterium. (*B*) Proportion of identified proteins that are classified as RBPs by Gerstberger et al. (2014), contain an RRM, or are directly annotated with the GO term “RNA binding.” (*C*) Venn diagram showing the overlap between known SR/SR-related proteins (Long and Caceres 2009), putative SR/SR-related proteins (this study), RBPs (only considering RBPs in either the known or putative SR/SR-related protein groups), and RRM-containing proteins (as identified by Pfam). (*D*) GO term analysis of new SR-related RBPs identified in this study (proteins outlined in red in panel *C*).

Previously, we developed an algorithm, LCD-Composer, to identify LCDs on the basis of customizable amino acid composition characteristics (Cascarina et al. 2021). We adopted an unbiased composition scanning approach to identify proteins with S/R-rich LCDs (herein referred to as “RS domains”) in the human proteome using LCD-Composer. Since RS domains can vary in their balance of S and R, we used a range of composition thresholds, starting with minimum composition thresholds of 20% S and 20% R within a 20-residue window, then increasing the minimum S and/or R content in 5% increments until all possible combinations were generated (see Materials and Methods). As expected, the least stringent composition criteria identify the greatest number of proteins with RS domains, with the frequencies decreasing as S or R composition thresholds increase (Fig. 1A). RS domains often cooccur with RRMs, and SR/SR-related proteins have classically been linked to RNA processing (Long and Caceres 2009). To explore the relationship between our composition thresholds and the ability of identified proteins to interact with RNA, we gathered a set of nonredundant human RNA-binding proteins (RBPs; see Materials and Methods). At low combined S + R composition thresholds, a low proportion of proteins are classified as RBPs (Fig. 1B). However, as the combined S + R composition threshold is increased, the proportion of SR/SR-related proteins also classified as RBPs progressively increases. The increase in the proportion of RBPs occurs at a faster rate as the R composition threshold is increased. However, increasing S content is also associated with a higher proportion of RBPs, even among domains with modest R enrichment, indicating that RS domains across a diverse range of S and R compositions are associated with RBPs. Analogous searches for lysine/serine-rich LCDs (KS domains) yield fewer proteins and less enrichment of RBPs (Supplemental Fig. S1), suggesting that these results are specific for RS domains.

In order to define a single set of SR/SR-related proteins, we selected a relatively high combined S + R composition threshold of 70% and generated a nonredundant set of 132 identified proteins. This combined threshold is such that >65% of the proteins identified are also classified as RBPs for all S + R composition criteria. It is also the highest threshold (among those tested) that captures >90% of the known SR/SR-related proteins (Supplemental Fig. S2). All windows passing these thresholds were then merged for each protein to generate the longest possible contiguous RS domain for each region. All identified RS domains are provided in Supplemental Table S1, along with additional data including cooccurrence with RRMs, whether the protein was previously classified as an SR/SR-related protein, SR/RS dipeptide frequencies, and PTM sites, as discussed below.

We compared our putative set of SR/SR-related proteins to the set of 52 known SR/SR-related proteins from Long and Caceres (Long and Caceres 2009). Using our strategy, all but three known SR/SR-related proteins are identified (Fig. 1C), including 45 known SR/SR-related proteins classified as RBPs and all four known SR-related proteins that are not RBPs yet still fit the definition of a noncanonical SR-related protein by Long and Caceres, indicating that our method is highly sensitive and capable of detecting both RBP and non-RBP SR/SR-related proteins. We identified an additional 83 proteins containing at least one RS domain of high S + R composition, 35 of which are also classified as RBPs. Furthermore, classical definitions of SR proteins often require the cooccurrence of at least one RRM (Manley and Krainer 2010). Nearly half of the SR/SR-related proteins defined by Long and Caceres, which included classical and nonclassical SR/SR-related proteins, contained an RRM identified by Pfam (Fig. 1C). However, in addition to the known SR/SR-related proteins, we identified 11 new SR-related proteins containing at least one RRM (Fig. 1C), further supporting the inclusion of these candidates as true SR-related proteins. Additionally, our 35 new SR-related RBPs are significantly associated with nuclear speckles and a variety of functions related to mRNA splicing and transport (Fig. 1D), nearly all of which (25 out of 27) are identical to functions significantly associated with the SR/SR-related protein set defined by Long and Caceres (Supplemental Table S2), supporting the inclusion of our new candidates in the SR-related protein family. A variety of splicing-related functions remain significantly associated with the new SR-related RBPs even when proteins with mixed-charge domains (which tend to localize to nuclear speckles and can include phosphorylated RS domains; Greig et al. 2020) or human homologs of mouse SR/SR-related proteins (Calarco et al. 2009) are excluded as well (Supplemental Fig. S3). Comparable sets of proteins with S-rich-only or R-rich-only domains are not significantly associated with classical SR/SR-related protein functions (Supplemental Tables S3, S4), indicating that these results are specific for proteins with RS domains.

Finally, when RS domains are identified with slightly lower combined S + R composition thresholds (≥60% S + R or ≥65% S + R), a greater number of functions are significantly enriched among the corresponding candidate SR-related proteins (Supplemental Tables S5, S6), which is likely due predominantly to the gain in statistical power associated with larger sample sizes. Indeed, the degree of enrichment associated with each function tends to be highest for the 70% S + R composition threshold (Supplemental Table S6) regardless of whether the enrichment reached statistical significance for the 70% threshold. This indicates both that the 70% S + R composition threshold yields slightly higher specificity with respect to expected functional categories, and that proteins identified at lower S + R composition thresholds may also be reasonable candidates for inclusion in the SR-related protein family.

Collectively, the high sensitivity and specificity of our method (as evidenced by the successful identification of all but three known SR/SR-related proteins and the relatively high enrichment of RBPs and RRM-containing proteins) suggest that these putative SR-related proteins have features consistent with both classical and nonclassical definitions of the SR/SR-related protein family.

### RS domain features of new SR-related proteins resemble those of known SR/SR-related proteins

**FIGURE 2.**
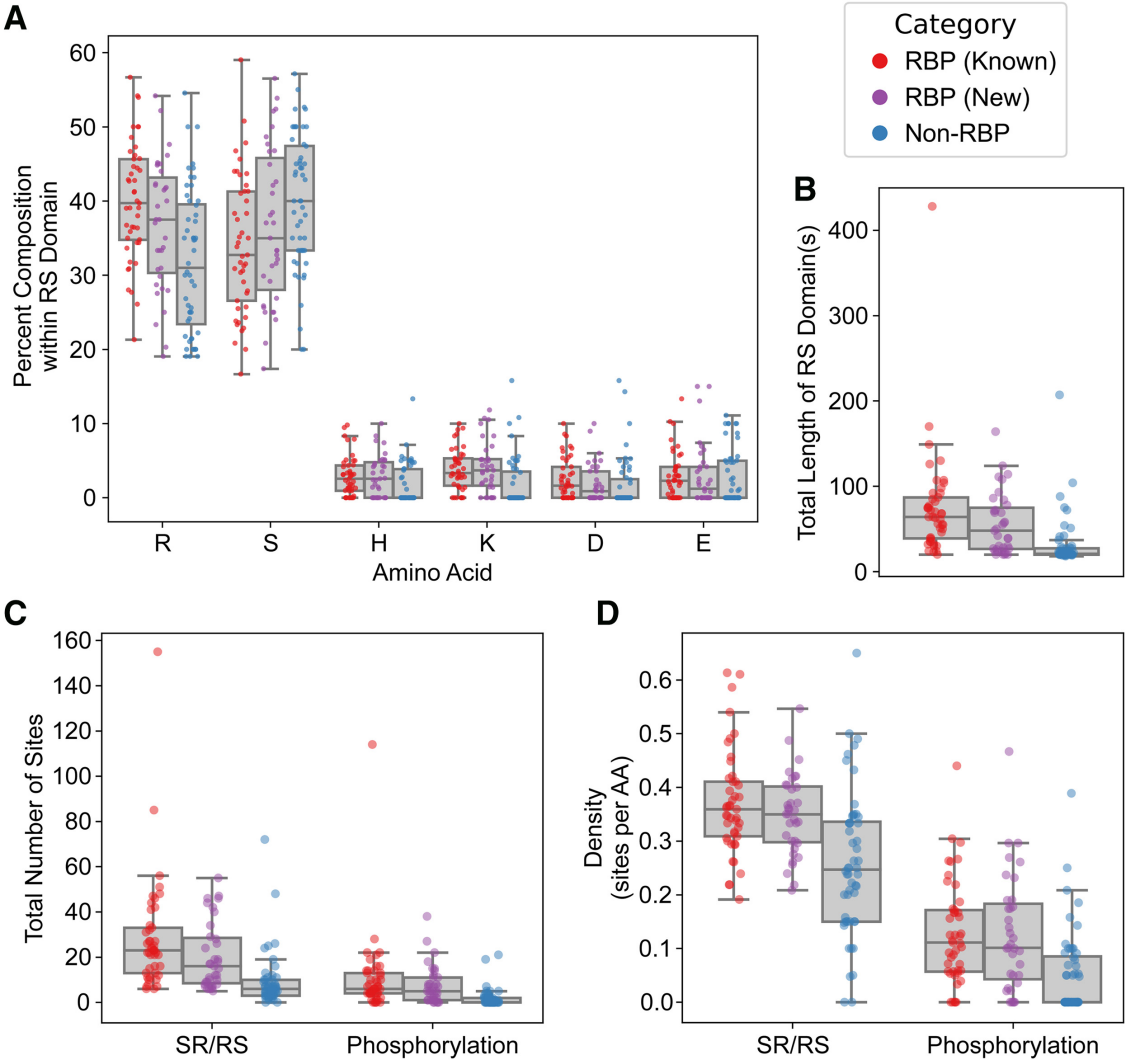
Sequence and PTM characteristics among RBP and non-RBP SR/SR-related proteins. (*A*) Percent composition within the RS domains of known SR/SR-related proteins, stratified into categories. “Known” SR/SR-related RBPs are from Long and Caceres (2009), “New” SR-related RBPs are the 35 proteins outlined in red in Figure 1C, and “Non-RBP” represents the 48 non-RBP SR-related proteins not found in the Long and Caceres data set and not classified as RBPs (see Fig. 1C). Composition analyses for all amino acids within RS domains can be found in Supplemental Figure S4. (*B*) Lengths of RS domains in known SR/SR-related RBPs, new SR-related RBPs, and SR-related non-RBPs. (*C*) Total number of SR and RS dipeptide sites and phosphorylation sites in RS domains of known SR/SR-related RBPs, new SR-related RBPs, and SR-related non-RBPs. (*D*) Density of SR/RS dipeptide sites and phosphorylation sites in known SR/SR-related RBPs, new SR-related RBPs, and SR-related non-RBPs.

Proteins with compositionally similar LCDs are often associated with specific sets of related functions even in the absence of primary-sequence similarity (Cascarina et al. 2021). Likewise, intrinsically disordered regions exhibit conservation of compositional and biophysical characteristics despite divergence in primary sequence (Zarin et al. 2019, 2021). Together, this suggests that compositional features are often directly (if not deterministically) linked to LCD function. To determine whether RS domains from our new SR-related protein candidates resemble those of known SR/SR-related proteins, we examined the compositional characteristics of the RS domains found in new and previously identified SR/SR-related proteins. The RS domains of the known SR/SR-related RBPs and (to a lesser extent) the new SR-related RBPs tended to have higher R content and lower S content relative to the RS domains of SR-related non-RBPs (Fig. 2A; Supplemental Table S1). Additionally, the known and new SR/SR-related RBPs also tended to have slightly higher H and K content in the RS domains than non-RBPs (though these differences are less pronounced), suggesting that these residues could also contribute to RNA binding affinity. Only weak differences are observed between RBPs and non-RBPs with respect to negatively charged residues (D/E; Fig. 2A) and other amino acids (Supplemental Fig. S4).

RS domains are often post-translationally modified, typically by phosphorylation, and a well-known family of SR protein kinases (SRPKs) preferentially modify SR/RS dipeptides (Giannakouros et al. 2011). To evaluate PTM and SR/RS dipeptide frequencies, we first examined the lengths of RS domains in RBP and non-RBP SR/SR-related proteins. RS domains from known and new SR/SR-related RBPs tend to be longer than those from non-RBPs (Fig. 2B), consistent with a previously observed correlation between RS domain length and splicing activity (Graveley et al. 1998). Correspondingly, RS domains from known and new SR/SR-related RBPs also tend to contain more total SR/RS dipeptides and more phosphorylation sites (Fig. 2C). However, when normalized based on total RS domain length, the known and new SR/SR-related RBPs also have a higher density of SR/RS dipeptides, though the phosphorylation site density does not differ substantially (Fig. 2D). Other types of PTMs are relatively rare within RS domains across all three categories (Supplemental Fig. S5).

Collectively, these observations indicate that our newly identified SR-related RBPs exhibit a number of shared characteristics and are distinct from SR-related non-RBPs. Differences in total length and SR/RS density between SR/SR-related RBPs and SR-related non-RBPs likely correspond to functional differences.

### Naturally occurring sequence variation frequently influences human RS domains

Alternative splicing commonly affects intrinsically disordered domains, LCDs, and repetitive protein regions (Romero et al. 2006; Haerty and Golding 2010; Buljan et al. 2013), although it is not clear whether this effect is uniform across different types of LCDs and disordered regions. RS domains play a direct physical role in splice site and branch point recognition (Shen and Green 2006). Additionally, naturally occurring protein isoforms can result from alternative promoter usage, alternative translation start sites, or ribosomal frameshifting. However, the degree to which RS domains themselves are included, excluded, or otherwise altered in isoforms of SR/SR-related proteins is currently unclear.

**FIGURE 3.**
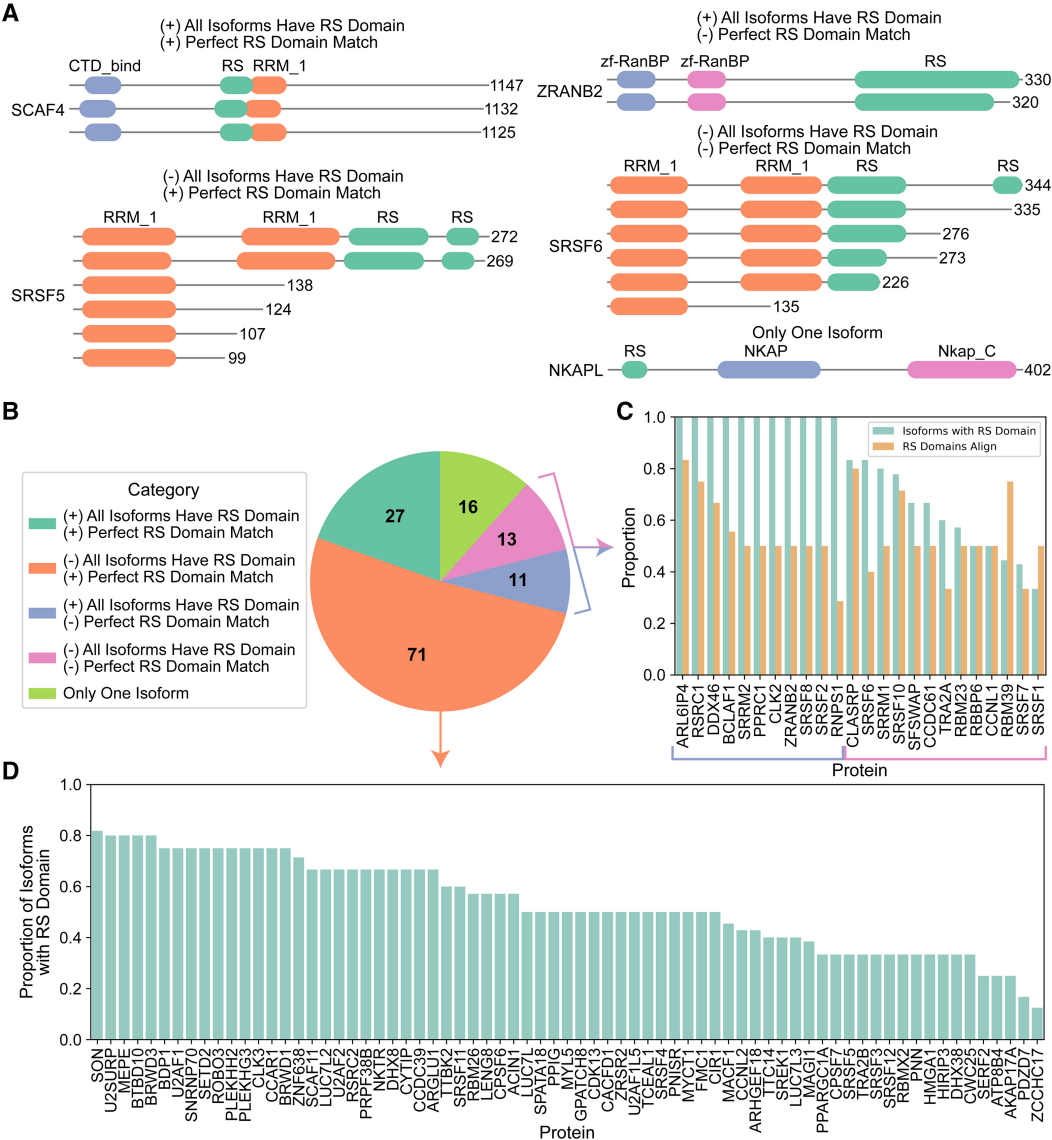
RS domains of human SR/SR-related proteins are frequently affected across isoforms. (*A*) Representative examples of sequence variation effects on RS domains among SR-related protein isoform sets based on: (1) whether all isoforms in the set contain an RS domain, and (2) whether all extant RS domains for an isoform set perfectly match each other. For these two criteria, (+) indicates isoform sets for which the criterium is true, whereas (−) indicates isoform sets for which the criterium is false. (*B*) Frequency analysis of sequence variation effects on human SR/SR-related protein isoform sets. (*C*) Proportion of isoforms that contain an RS domain and proportion of existing RS domains that perfectly align for proteins with RS domains that do not perfectly match (24 proteins in panel *B*). (*D*) Proportion of isoforms that contain an RS domain for all isoforms where the existing RS domains perfectly align (71 proteins in panel *B*).

To explore whether RS domains commonly differ across isoforms for each SR/SR-related protein, we repeated the S + R composition scanning on a proteome containing all known human protein isoforms (see Materials and Methods). In total, 369 isoforms mapping to 138 unique genes contain at least one RS domain (Fig. 3; Supplemental Table S7)—a slight increase relative to our previous set due to RS domains that are present in at least one alternative isoform but absent in the representative isoform. 16 of the identified proteins have only one isoform in the human proteome. For the remaining sets of isoforms, we evaluated: (1) whether all isoforms associated with each protein contain at least one RS domain, and (2) whether all identified RS domains for a set of isoforms are perfect sequence matches, ignoring isoforms that did not contain an RS domain (Fig. 3A,B). Only 27 proteins have identical RS domains present in all isoforms. Eighty four proteins have at least one isoform lacking an RS domain (Fig. 3B–D); of these, 13 also show variation among the existing RS domains (Fig. 3B,C), which could be due either to inclusion/exclusion of a second RS domain, or to variation in the sequences of the RS domains. For an additional 11 proteins, all of the isoforms contain at least one RS domain, but some of the RS domains differ. The canonical SR proteins (SRSF1–SRSF12) exhibit particularly striking variation across isoforms: for all 12 proteins, there exists at least one isoform containing a truncation or complete omission of one or more RS domains (Supplemental Fig. S6). Thus, natural sequence variation frequently influences the presence, length, and/or sequence of RS domains among SR/SR-related proteins, which may broadly alter the splicing activity of affected proteins and result in complex cascades of splicing regulation (Gueroussov et al. 2015, 2017; Deshaies et al. 2018; Fratta and Isaacs 2018).

### New SR-related proteins localize to nuclear speckles, preferentially bind mRNA, and are involved in RNA metabolism

A wealth of data pertaining to the localization and functions of RBPs has recently been published as part of the ongoing ENCORE project (Van Nostrand et al. 2020). We cross-referenced our set of known and new SR/SR-related RBPs with available ENCORE resources. Despite the monumental scale of the ENCORE project, only 20 known SR/SR-related proteins (∼41% of the known SR/SR-related proteins detected) and six new SR-related proteins (∼17% of new SR-related RBPs) have been studied thus far, and only in select experiments (Supplemental Fig. S7A). Additionally, based on a systematic evaluation of the literature and manual annotation of functions (Van Nostrand et al. 2020), the new SR-related proteins only have one annotated function on average, whereas the known SR/SR-related proteins have two functions on average (Supplemental Fig. S7B). Together this suggests either that new SR-related proteins are more specialized proteins with fewer functions, or that these candidates are understudied relative to known SR-related proteins.

**FIGURE 4.**
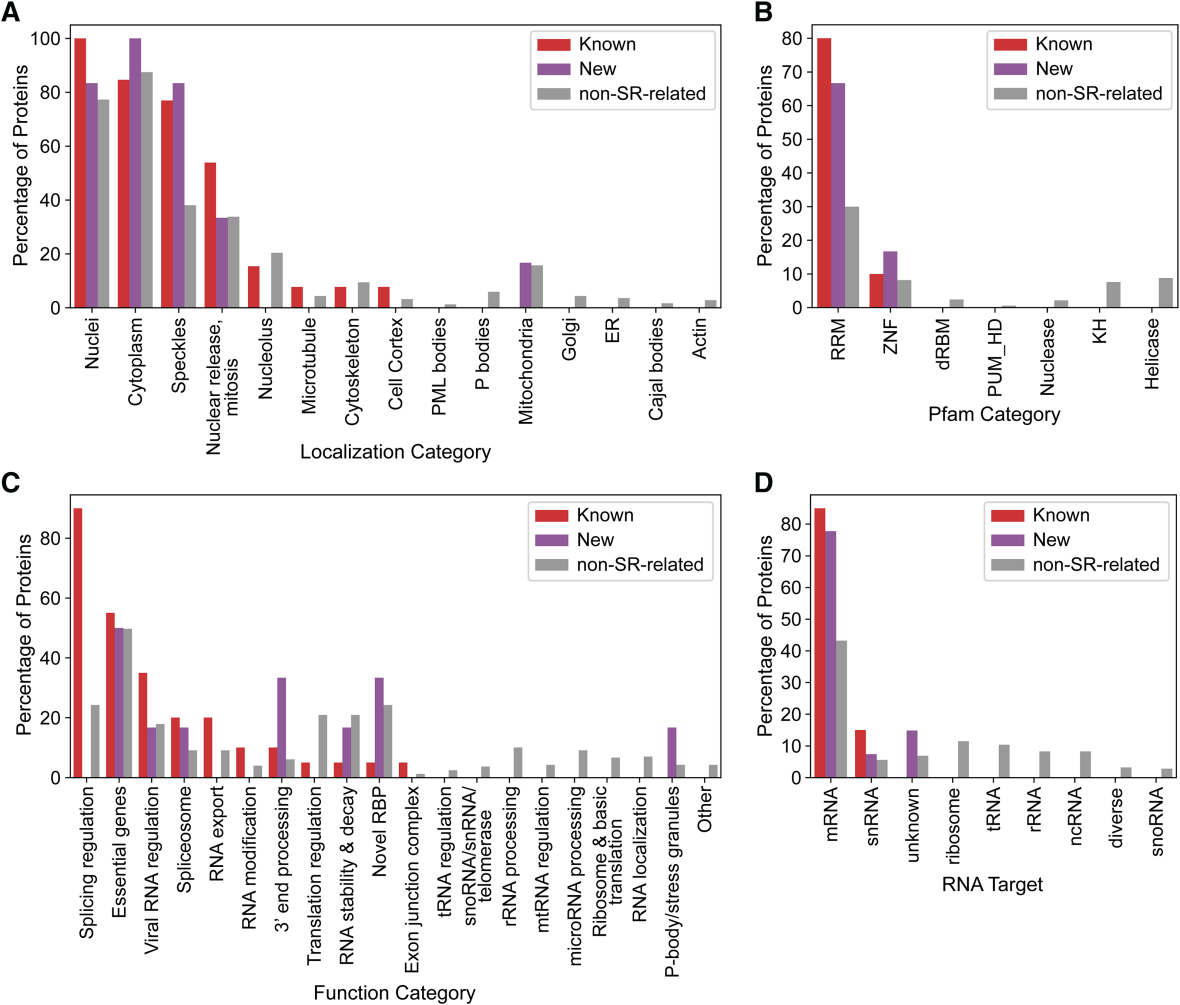
New SR-related RBPs localize to nuclear speckles and participate in mRNA processing. (*A*) Percentage of proteins in each category with experimentally verified localization to specific subcellular compartments. (*B*) Predicted Pfam annotations assigned to available proteins from each category. (*C*) Percentage of proteins with literature-derived function annotations assigned to each RBP. (*D*) Percentage of proteins assigned to each RNA target category, derived from a literature-based consensus of the type of RNA bound by each RBP.

We took advantage of these smaller sample sizes to manually compare these known and new SR/SR-related proteins in greater depth (Table 1; Fig. 4A–C). Like the known SR/SR-related proteins, the new SR-related proteins are strongly associated with localization to the nucleus, cytoplasm, and nuclear speckles (Fig. 4A) and have similarly high levels of coinciding RRMs (Fig. 4B). In contrast, a relatively low proportion of non-SR-related RBPs localize to nuclear speckles or contain an RRM (Fig. 4A,B). Based on literature-derived functional annotations, the new SR-related proteins exhibit less-prominent association with splicing regulation (despite their presence in nuclear speckles) and are instead associated with an assortment of RNA metabolism-related functions, including 3′ end processing, RNA stability and decay, P-bodies/stress granules, and viral RNA regulation (Fig. 4C), but this may be affected by the relatively small number of functions currently known for the new SR-related proteins (Supplemental Fig. S7A,B). A larger set of literature-derived annotations (Gerstberger et al. 2014), which includes 40 known and 27 new SR/SR-related proteins, indicates that both groups exhibit a similarly high preference for binding mRNA relative to the typical RNA-binding preferences of non-SR-related proteins (Fig. 4D). Finally, the known and new SR/SR-related proteins affect the expression of similar numbers of genes and are predicted to bind to large numbers of unique transcripts relative to non-SR-related proteins, though the effect of the new SR-related proteins on all forms of alternative splicing more closely resembled that of non-SR-related proteins (Supplemental Fig. S7C–E).

**TABLE 1.**
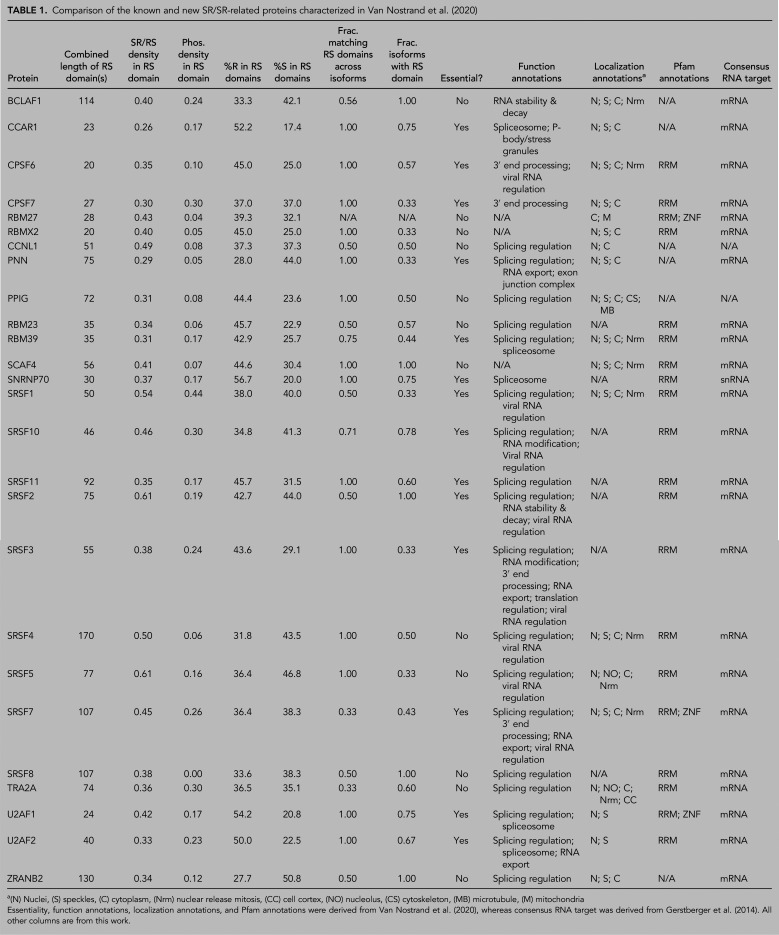
Comparison of the known and new SR/SR-related proteins characterized in Van Nostrand et al. (2020)

Although these sample sizes are severely limited, the available data further support a role for the new SR-related RBPs in nuclear speckles and mRNA metabolism via direct physical interaction with mRNA.

### SR-related proteins are associated with DEAD-box helicases but not RRMs across archaea, bacteria, and eukaryotes

**FIGURE 5.**
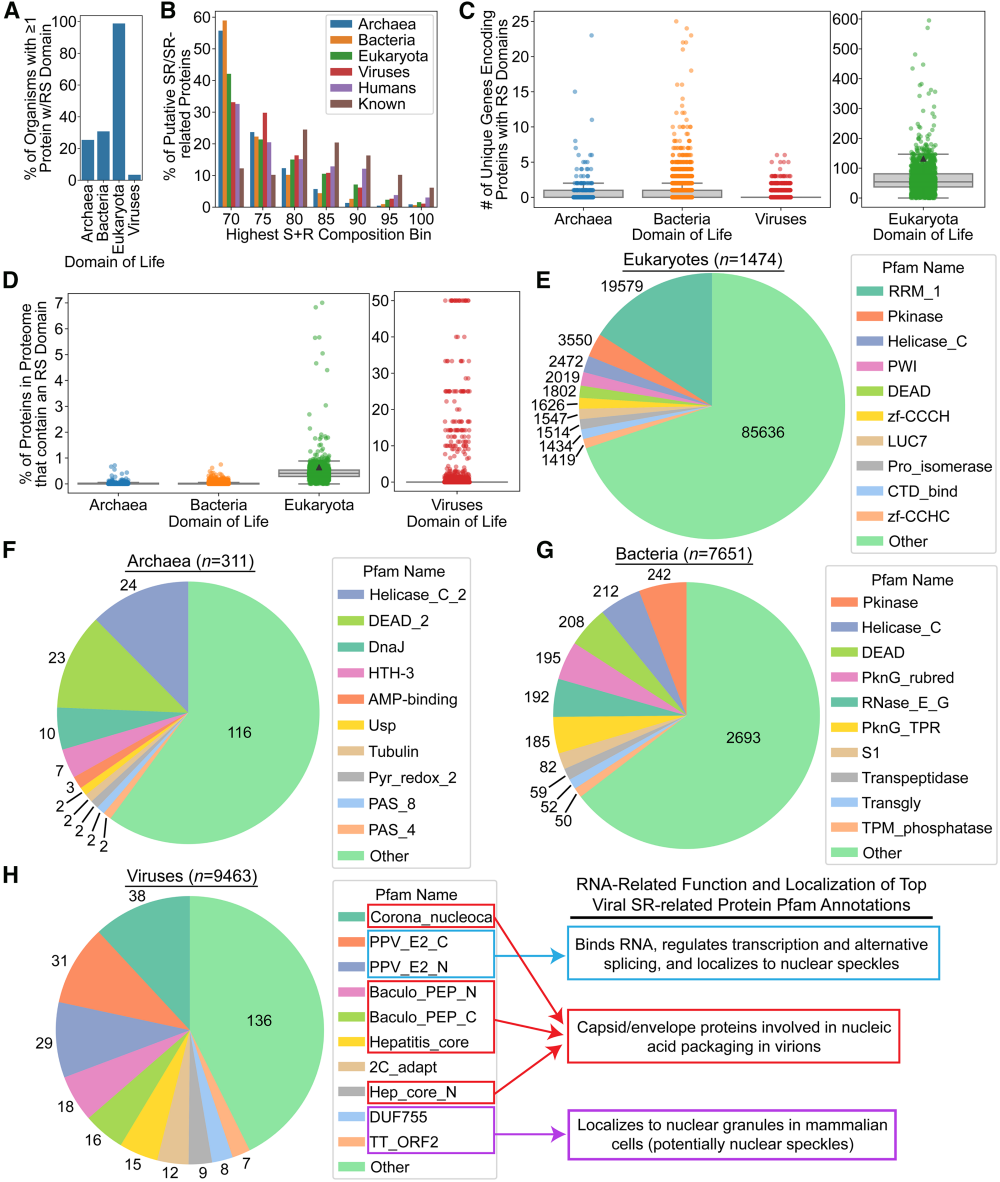
Frequencies of SR/SR-related proteins and Pfam annotations across four domains of life. (*A*) The percentage of proteomes from each domain of life containing at least one SR/SR-related protein. (*B*) The percentage of SR/SR-related proteins (*y*-axis) from each domain of life achieving each maximum S + R composition within a 20-residue scanning window (*x*-axis). (*C*) For each organism, the total number of SR proteins was calculated and plotted. The black triangle on the Eukaryota plot represents the number of unique proteins in the human proteome (excluding isoforms). (*D*) The percentage of proteins containing an RS domain among all organisms with at least one SR/SR-related protein. The top 10 most common Pfam annotations and their frequencies are indicated for eukaryotes (*E*), archaea (*F*), bacteria (*G*), and viruses (*H*). Parenthetical values in *B*–*E* indicate the total number of organisms analyzed.

Our focus on human SR/SR-related proteins, which have been studied extensively, allowed us to establish and validate our RS domain search method. Using identical search criteria (S + R composition ≥ 70%), we identified all RS domains across all known reference proteomes available from UniProt (Supplemental Tables S8–S11). Nearly all eukaryotes have at least one SR/SR-related protein, whereas only ∼25%–30% of archaea and bacteria have an SR-related protein (Fig. 5A). Eukaryotic RS domains are slightly more skewed toward very high maximum S + R percent composition bins (Fig. 5B), but similar distributions of R and S levels within the full length RS domains are observed across all four domains of life (Supplemental Fig. S8). Furthermore, RS domains from all four domains of life exhibit minor secondary preferences for A, G, P, and T, with an additional slight preference for non-R charged residues specifically among eukaryotic RS domains. RS domains are abundant in most eukaryotic organisms, but relatively rare among archaea, bacteria, and viruses (Fig. 5C,D), consistent with the reported absence of spliceosome-dependent mRNA splicing in noneukaryotic organisms (Vosseberg and Snel 2017). However, the rarity of RS domains in these domains of life does not necessarily imply functional insignificance.

RRMs are substantially more common in eukaryotes than other forms of life due, at least in part, to their prevalence among highly diversified splicing factors. Therefore, we explored whether any other types of domains were commonly associated with SR/SR-related proteins across all domains of life. For each protein, a nonredundant list of Pfam domains was collected (Supplemental Tables S8–S11). Then, for all unique types of Pfam domains, the frequency of that domain across SR/SR-related proteins was calculated separately for each domain of life. Figure 5E–H indicates the 10 most frequent Pfam domains for each domain of life. Nearly all of the most frequent Pfam annotations are significantly more enriched among SR/SR-related proteins than among comparable sets of proteins with S-rich-only or R-rich-only domains (Supplemental Table S12), indicating that these results are specific for SR/SR-related proteins. Interestingly, DEAD/DEAD_2 and Helicase_C/Helicase_C_2 were among the top five annotated domains across archaea, bacteria, and eukaryotes. These domains often occur within the same protein and are typically associated with RNA helicases, though they can act on other nucleic acids as well. Additionally, these SR-related helicases exhibit remarkably consistent protein domain architectures (Supplemental Fig. S9A–C), frequently recover orthologs in many other organisms of that domain of life (e.g., 49.8% of archaea), and the orthologs often contain S/R-rich regions (Supplemental Fig. S9D), suggesting that the RS domains are functional elements within these proteins. In contrast, protein kinase domains (“Pkinase”) were the second most frequent domain (behind RRMs) in SR/SR-related proteins from eukaryotes yet the rate of occurrence did not differ significantly from its rate of occurrence among S-rich-only proteins (Supplemental Table S12), highlighting an example of a domain that is common but not specifically enriched among SR/SR-related proteins. Finally, in addition to RRMs, eukaryotic SR/SR-related proteins also often have PWI domains, which are RNA-binding domains involved in RNA processing (though PWI domains are ∼10-fold less abundant than RRMs).

Collectively, these observations highlight common, nonsplicing functions associated with SR/SR-related proteins across organisms.

### Viral SR-related proteins are associated with nucleic acid packaging and processing

Viruses are unique in that they rely on host-organism proteins to carry out many molecular functions and express their own proteins in the context of the host intracellular environment. Consequently, viruses likely do not have a full complement of splicing factors, but may express proteins that influence splicing, as observed for many eukaryotic viruses (Ashraf et al. 2019; Boudreault et al. 2019). Additionally, most viral lifecycles effectively revolve around nucleic acid replication, processing, and packaging.

The most common Pfam domains are linked to RNA packaging across multiple types of viruses (Fig. 5H) and are specifically enriched among SR-related proteins compared to S-rich-only or R-rich-only proteins (Supplemental Table S12). These domains are found in proteins such as coronavirus nucleocapsid proteins (“Corona_nucleoca”), baculovirus polyhedron envelope proteins (“Baculo_PEP_N” and “Baculo_PEP_C”), and hepatitis capsid proteins (“Hepatitis_core” and “Hep_core_N”). The nucleocapsid (N) protein of SARS-CoV-2 was identified previously by a general LCD search (Cascarina and Ross 2020); it is among our SR-related candidates here (Supplemental Table S11) and contains an amino-terminal RNA-binding domain, which is a common feature among coronavirus nucleocapsid proteins (McBride et al. 2014). Phosphorylation of the RS domain in coronavirus N proteins has been proposed as a common regulatory mechanism for capsid assembly and disassembly (Nikolakaki and Giannakouros 2020). A number of studies support a role for the RS domain of the SARS-CoV-2 N protein in modulating liquid–liquid phase separation, RNA binding, genomic viral RNA packaging, and recruitment to stress granules in mammalian cells: these activities are also regulated by host cell SR kinases, though it should be noted that other studies implicate non-RS regions of the N protein as critical domains for stress granule recruitment and phase separation (for review, see Cascarina and Ross 2022). Strikingly, about half of the N proteins from distinct coronaviruses contain an RS domain that meets or exceeds our favored 70% S + R composition threshold, nearly all of the remaining coronavirus N proteins still contain a region enriched in S + R (Supplemental Fig. S10A), and the RS domain always occurs in the central region of the protein (Supplemental Fig. S10B).

The papillomavirus E2 protein (“PPV_E2_N” and PPV_E2_C”) was also common among viral SR-related proteins and is associated with a variety of RNA processing functions. For example, the E2 protein of various human papillomaviruses possesses all of the hallmark features and behaviors of classical SR-related proteins, including RNA binding; regulation of transcription and alternative splicing; localization to nuclei and nuclear speckles (Graham 2016); and the presence of an RS domain (Supplemental Table S11). Additionally, the torque teno virus ORF2 protein, which tends to contain both “DUF755” and “TT_ORF2” domains, exhibits a granular localization pattern in nuclei consistent with nuclear speckles when expressed in mammalian cells in vitro and is thought to have a role in mRNA splicing (Mueller et al. 2008), though this requires more detailed experimental validation.

## DISCUSSION

A variety of approaches have been utilized in prior attempts to identify SR and SR-related proteins from among whole proteomes (Boucher et al. 2001; Calarco et al. 2009; Califice et al. 2012; Greig et al. 2020). While a consensus has been reached regarding a definition for core members of the SRSF protein family (Zahler et al. 1992; Manley and Krainer 2010), the diversity of approaches to identify SR-related proteins reflects a lack of consensus on biologically relevant criteria for inclusion into the SR-related protein family. At a minimum, the defining feature of SR-related proteins is the presence of one or more S/R-rich LCDs. However, the precise composition thresholds for S and R enrichment appropriate for defining RS domains (or any type of LCD) are difficult to rationally define and are, to some degree, subjective by nature.

With these considerations in mind, we adopted a composition scanning approach, coupled with pre-existing knowledge of RNA binding proteins, to rationally identify SR/SR-related proteins. We demonstrate that this approach is highly sensitive, detecting 49 of the 52 known SR/SR-related proteins, yet specific enough to result in enrichment of functions typically associated with SR/SR-related proteins among our new candidate proteins: we identified 35 completely new SR-related proteins associated with RNA binding activity, nuclear speckle recruitment, RNA processing, and RNA trafficking. Additionally, a recent study found that the SON and SRRM2 proteins (which have three and 10 distinct RS domains, respectively; Supplemental Table S1) are essential for nuclear speckle formation (Ilık et al. 2020), though only SRRM2 was classified as an SR-related protein in Long and Caceres (2009), and neither protein qualifies as a canonical SR protein (Manley and Krainer 2010). Since (1) our approach identifies many SR/SR-related proteins already defined in previous studies, and (2) we detected many relevant functional associations among SR/SR-related proteins unique to our study, we believe that our method is one of many reasonable approaches and should be viewed as complementary to (rather than in competition with) prior studies. However, it is also worth noting that additional SR-related proteins identified at slightly lower S + R composition thresholds still contain a high proportion of RBPs (Supplemental Table S5) and exhibit enrichment of similar functions (albeit often to lesser degrees; Supplemental Table S6) compared to the SR-related proteins identified with a 70% combined S + R composition threshold. Indeed, the three known SR/SR-related proteins that are not detected using our 70% combined S + R threshold are detected at slightly lower S + R thresholds (65%, 60%, and 55%, respectively). Therefore, many proteins identified at lower S + R composition thresholds could arguably be included in the SR-related protein family. Similarly, the RS domains of some SR/SR-related proteins may extend beyond the boundaries of those defined using the 70% S + R threshold but with lower S + R content in the extension regions: in such cases, the region passing the stringent 70% S + R threshold may be viewed as the “core” RS domain, while lower thresholds could be chosen (based on a particular research question) to define extensions of this core domain. More extensive lists of proteins and their maximum S + R composition within a 20-residue window are provided as additional resources for those interested in exploring the use of alternative composition thresholds or window sizes (Supplemental Tables S13–S16).

Phosphorylation is a prominent regulator of RS domains. RS domains among our new SR-related proteins are heavily phosphorylated regardless of whether they are classified as RBPs, consistent with PTM-dependent regulation of their activity in both canonical and noncanonical functions (Giannakouros et al. 2011). Recently, increased phosphorylation of RS domains was shown to enhance LLPS and recruitment to nuclear speckles (Greig et al. 2020)—a membraneless compartment with liquid-like properties—whereas dephosphorylation was associated with mislocalization, oligomerization, and aggregation of SR/SR-related proteins (Kundinger et al. 2021). Therefore, widespread phosphorylation of RS domains may represent a remarkably generic yet potent mechanism to regulate the localization, solubility, and activity of SR/SR-related proteins, though likely in conjunction with unique, site-specific effects.

Given the strong association between SR/SR-related proteins and mRNA splicing, as well as a direct role for RS domains in splice site recognition and specificity (Shen and Green 2006), it is remarkable how often RS domains differ among isoforms of SR/SR-related proteins themselves: of the 138 isoform sets corresponding to SR/SR-related proteins, ∼80% of the isoform sets have at least one isoform whose RS domain is missing or altered (Fig. 3). In principle, regulation of RS domains by alternative splicing could lead to rather complex splicing logic, which could then permit exquisite control of splicing programs in a time-, condition-, or tissue-specific manner. However, RS domains in splicing factors are not always strictly required for splicing activity (Zhu and Krainer 2000): it is equally possible that inclusion or exclusion of a small number of RS domains could act as master regulators or gatekeepers of splicing programs, analogous to a recently proposed model involving splicing of the mouse Srsf10 protein (Meinke et al. 2020). Alternatively, RS domains could play a more auxiliary role in splicing, perhaps only enhancing splicing activity or splice site specificity.

A number of proteins can be classified as SR-related proteins even though they lack RNA binding activity and do not seem to be involved (at least directly) in RNA splicing (Long and Caceres 2009). Previous studies have suggested that SR/SR-related proteins can also be involved in a variety of peripheral functions unrelated to RNA splicing, including nucleocytoplasmic shuttling, translation, chromatin organization, cell cycle regulation, and metabolism (Long and Caceres 2009; Shepard and Hertel 2009; Zhong et al. 2009; Giannakouros et al. 2011; Wagner and Frye 2021; Slišković et al. 2022). Given the overwhelming abundance of RNA splicing proteins among SR/SR-related proteins and the diversity of nonsplicing functions among SR/SR-related proteins, these peripheral functions would likely not be enriched enough to reach statistical significance in GO term analyses, but may nevertheless suggest subclasses of SR-related proteins associated with specific processes. Interestingly, in addition to the new SR-related RBPs, nearly half of the new SR-related non-RBPs (23/52) are still associated with the nucleoplasm, and ∼11% of the proteins (6/52) are associated with nuclear speckles based on existing GO annotations (Supplemental Table S17). Though neither of these associations reach statistical significance after multiple-test correction, when coupled with the recent finding that RS domains can associate with nuclear speckles upon phosphorylation (Greig et al. 2020), it may suggest a weak link between our new SR-related non-RBPs and recruitment to the nucleus and nuclear speckles. Furthermore, incomplete functional annotation may actually underestimate the number of proteins associated with nuclear speckles and splicing: for example, the NKAP protein (UniProt ID: Q8N5F7) was shown to localize to nuclear speckles, interact with spliceosomal proteins, and influence mRNA splicing (Burgute et al. 2014), yet it is currently not annotated with any of those functions (Supplemental Table S18). Furthermore, five of the six new SR-related proteins evaluated in Van Nostrand et al. (2020) exhibit experimentally observed localization to nuclear speckles, yet none of these proteins were annotated as such in the gene ontology. Collectively, this suggests a role for at least a subset of additional SR-related proteins (both RBP and non-RBP) in alternative splicing or nuclear speckle regulation. However, we also reemphasize that the well-studied connection between RS domains and splicing should not overshadow a role for RS domains in other cellular processes. RS domains exhibit remarkable, phosphorylation-regulated structural plasticity (Hamelberg et al. 2007) and can function as nuclear transport signals (Maertens et al. 2014)—presumably generic functions that could be coopted by a variety of proteins. It is also possible that non-RBP SR-related proteins could serve as functional bridges between nuclear speckles/splicing and a variety of other cellular processes (Supplemental Fig. S11): subsets of these proteins also contain domains typically associated with DNA/chromatin binding (e.g., “bromodomain,” “SET,” “NAP,” “Myb_DNA-binding,” “CHZ,” “zf-C2H2”), kinase/signal transduction activity (e.g., “Pkinase,” “Guanylate_kin,” “Rho-GEF”), or microtubule/cell cycle regulation (e.g., “INCENP_N,” “Cyclin_N,” “Cyclin_C,” “PDZ”).

Using our composition criteria for RS domains, we identified SR-related proteins among a set of reference proteomes representing ∼18,900 organisms. Spliceosome-dependent mRNA splicing is not currently believed to exist in any known bacterial or archaeal organisms (Vosseberg and Snel 2017), so SR-related proteins in noneukaryotes are expected to have functions unrelated to splicing. Although SR-related proteins are rare in archaea and bacteria relative to eukaryotes, existing SR-related proteins in these three domains of life suggest a link between RS domains and proteins containing DEAD-box, helicase, or kinase domains. DEAD-box domains and helicase domains often cooccur in the same protein. It is tempting to speculate that RS domains had an original role in, for example, RNA helicase activity before being coopted by the litany of other splicing factors now containing RS domains in many eukaryotes. More broadly, this suggests that these SR-related protein functions are more universal across all life forms, even if RRMs and splicing-related functions are now the dominant associations in eukaryotes.

Finally, given their unique mode of replication, viruses appear to utilize RS domain-containing proteins for slightly different functions: namely, nucleic acid packaging and processing. Emerging research on SARS-CoV-2 supports a role for the RS domain of the nucleocapsid protein in mediating protein self-assembly and liquid–liquid phase separation, which is thought to influence host cell stress response, protein–protein interaction, protein–RNA interaction, viral genomic RNA packaging, and host cell translation regulation (Cascarina and Ross 2022). However, RS domains are only present in a relatively small subset of viruses, and a variety of viral genome packaging proteins lacking RS domains appear to undergo liquid–liquid phase separation as part of the nucleic acid condensation and encapsulation process (Cascarina and Ross 2020). Therefore, RS domains may be one of many types of LCDs contributing to the self-assembly of capsid proteins and viral nucleic acids, and viral RS domains may have molecular roles outside of nucleic acid packaging (as observed for the papillomavirus E2 protein).

In summary, our unbiased S + R composition search successfully identified both known and new SR/SR-related proteins with strong links to classical SR/SR-related protein localization and function in humans, and uncovered SR-related proteins and their associated functions in noneukaryotic organisms.

## MATERIALS AND METHODS

### Data acquisition and processing

The reference human proteome was downloaded from the UniProt KB website on 6/18/2020. Proteomes for all other archaeal, bacterial, and eukaryotic organisms were downloaded from the UniProt FTP server (ftp://ftp.uniprot.org/pub/databases/uniprot/) on 8/21/2020. All virus proteomes were downloaded from the same site on 8/23/2020-8/24/2020. The organism corresponding to UniProt ID UP000292173_1906665 was misclassified as an archaeon and, therefore, excluded from all analyses. Human RBPs were defined as proteins in Gerstberger et al. (2014), proteins directly annotated as “RNA-binding” (GO id:0003723), or proteins that contained one or more RRMs as defined by Pfam. For analyses of human SR/SR-related protein isoforms, all isoforms annotated as readthrough products or as incomplete protein fragments were removed prior to analysis. A database of PTMs in human proteins was downloaded from ActiveDriverDB (https://www.activedriverdb.org/download/; Krassowski et al. 2018) on 1/24/2021. For each type of PTM (e.g., phosphorylation, acetylation, etc.), all PTM sites from the ActiveDriverDB were mapped to their respective locations in the corresponding protein. PTM sites were then cross-referenced with RS domain boundaries to determine the number of PTMs and the PTM density within RS domains. The GO annotation file for human proteins was downloaded from ftp://ftp.ebi.ac.uk/pub/databases/GO/goa/ on 2/27/2020. The gene ontology file was downloaded from http://geneontology.org/ on 2/27/2020.

Data representing the ENCORE project (Van Nostrand et al. 2020) were mapped to our set of new and known SR/SR-related proteins. Only two of our new SR-related RBPs were represented in the eCLIP data sets (Supplemental Fig. S7A) characterizing specific transcripts bound by each RBP. Therefore, we used the downloadable RNAct database (Lang et al. 2019) of predicted RNA–protein interactions—which is based on the *cat*RAPID method (Bellucci et al. 2011) and validated on existing ENCORE data (Lang et al. 2019)—to estimate the number of transcripts bound by each RBP (Supplemental Fig. S7E). Only high-confidence RBP–RNA interactions (here defined as having a *z*-score ≥1 based on the distribution of scores for characterized ENCORE proteins; Lang et al. 2019) were evaluated.

### Identification of RS domains

Protein sequences were scanned for RS domains using LCD-Composer version 1.0 (https://github.com/RossLabCSU/LCD-Composer) with default parameters. Human protein sequences were scanned with a range of S and R composition thresholds, with an absolute minimum of 20% S and 20% R. Minimum S and R composition thresholds were increased in 5% increments until all possible combinations of S and R composition thresholds were used. This process was also performed in an identical manner to search for KS domains in the human proteome. To generate a single set of nonredundant SR/SR-related proteins, all proteins with at least one domain where the S + R composition exceeded 70% were pooled. All other proteomes were scanned using only S and R composition thresholds for which the sum was at least 70%, and the resulting proteins were pooled.

A two-stage process was used to identify sets of S-rich-only and R-rich-only proteins comparable to the SR/SR-related protein set based on the average S and R compositions among RS domains (37.6% and 35.5%, respectively). First, the human proteome was scanned using a 35% minimum composition threshold and 40% maximum composition threshold for S or R, with the additional constraint that the domains contain 0% R or 0% S, respectively. In order to achieve protein sample sizes comparable to the SR/SR-related protein set, the resulting S-rich-only or R-rich-only LCDs were further filtered such that the S or R composition closely matched the average S or R composition of the human RS domains (i.e., S composition between 37%–38%, or R composition between 35%–37%). These criteria result in *n* = 181 S-rich-only human proteins and *n* = 143 R-rich-only human proteins.

### Statistical analysis of domain and function annotations

Pfam annotations for all proteins were determined using the pfamscan.py script with default parameters, downloaded from https://www.ebi.ac.uk/seqdb/confluence/display/JDSAT/PfamScan+Help+and+Documentation on 2/9/2021 (Madeira et al. 2019; Mistry et al. 2021). For calculation of Pfam frequencies, Pfam annotations were only counted once per protein to prevent overrepresentation of Pfam domains that tend to occur multiple times in a single protein. When comparing SR/SR-related proteins to S-rich-only or R-rich-only proteins, the rate of occurrence for each of the top 10 Pfam annotations among SR/SR-related proteins was compared to its rate of occurrence among S-rich-only or R-rich-only proteins using a two-sided Fisher's exact test. Proteins appearing in both the SR/SR-related set and the appropriate comparison set (either S-rich-only or R-rich-only proteins) were removed prior to analysis. *P*-values were adjusted within each protein set and domain of life to account for multiple-hypothesis testing using the Holm–Šidák correction method. Odds ratios indicated by “N/A” represent cases for which the Pfam annotation did not occur among the comparison group. For Pfam analysis of the 12 canonical SR proteins, all protein isoforms (including those not containing an RS domain by our criteria) were analyzed using the Pfam server. RS domains within these proteins were defined as regions with ≥70% combined S + R composition, with the exception of the RS domain of SRSF9 (indicated as RS*), which could only be detected with a 60% combined S + R minimum composition. For domain mapping of SR-related helicases and coronavirus nucleocapsid proteins, the normalized distance of a domain from the protein amino terminus was defined as the starting position of the domain divided by the total length of the protein.

GO term enrichment analyses (Ashburner et al. 2000; Carbon et al. 2021) were performed using GOATOOLS version 1.0.2 (https://github.com/tanghaibao/goatools) with default parameters (Klopfenstein et al. 2018). GO terms directly assigned to the NKAP protein (UniProt ID: Q8N5F7) were collected from the human gene annotation file. When comparing GO terms significantly enriched among protein sets identified using a range of S + R composition thresholds (60%, 65%, and 70%), for each GO term significantly enriched in any of the three analyses, the degree of GO term enrichment was calculated as the natural logarithm of the odds ratio (i.e., the odds that a protein with the associated function is an SR/SR-related protein divided by the odds that a protein with the associated function is not an SR/SR-related protein). All conclusions relating to statistical enrichment of GO terms were based on Šidák-corrected *P*-values to account for multiple-hypothesis testing.

### Defining SR-related protein orthologs

Orthologs of SR-related helicases in archaea were identified using the “reciprocal best hit” method in conjunction with BLAST (v2.10.1) searches for each SR-related helicase in each organism. Briefly, for each BLAST search, the protein identified as the closest match for each SR-related helicase was used as a query protein for the reciprocal search. Matches were only considered reciprocal best hits if the original SR-related helicase was recovered as the best hit in the reciprocal search and the *E*-value for both searches was less than 0.05. For all BLAST searches, RS domains in the query sequence were masked prior to each search to ensure that they did not contribute to a bias toward recovering the SR-related helicase as the reciprocal best hit. For downstream analyses, all reciprocal best hits were pooled, parsed into bins based on the maximum S + R composition achieved in a 20-residue window for each protein, and compared to the complete set of all other proteins derived from archaeal proteomes. Coronavirus nucleocapsid proteins were manually recovered from the 83 coronavirus proteomes represented in our data set: the maximum S + R compositions for these proteins were then compared to the maximum S + R compositions of nonnucleocapsid proteins as described above.

## DATA DEPOSITION

All code required to reproduce the data in this article are available at https://github.com/RossLabCSU/RNA2022.

## SUPPLEMENTAL MATERIAL

Supplemental material is available for this article.

## Supplementary Material

Supplemental Material

## Figures and Tables

**Sean Cascarina:**
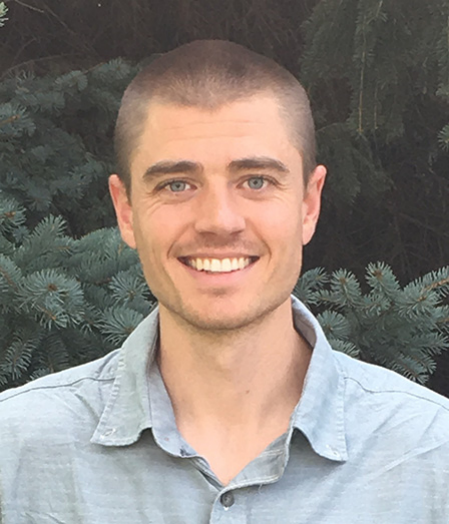


## MEET THE FIRST AUTHOR

***Meet the First Author(s)* is a new editorial feature within *RNA*, in which the first author(s) of research-based papers in each issue have the opportunity to introduce themselves and their work to readers of *RNA* and the RNA research community. Sean Cascarina is the first author of this paper, “Expansion and functional analysis of the SR-related protein family across the domains of life.” Sean is a biochemist and bioinformatician in the Biochemistry and Molecular Biology department at Colorado State University.**

**What are the major results described in your paper and how do they impact this branch of the field?**

Eukaryotes contain a number of proteins with Ser/Arg-rich low-complexity domains (referred to as “RS domains”), which constitute two similar protein families known as SR proteins and SR-related proteins. These proteins are often involved in mRNA splicing and RNA metabolism, though many have other functions as well. In our study, we used a bioinformatic tool to identify new SR-related proteins by implementing an unbiased composition scanning approach. Based on our data and recently published work, many of these new SR-related proteins exhibit hallmark biological activities of known SR/SR-related proteins, including extensive post-transcriptional and post-translational regulation affecting the RS domain, biological functions shared with known SR/SR-related proteins, and localization to the same subcellular compartments (nuclei and nuclear speckles in particular). Additionally, we showed that some noneukaryotic life forms also contain SR-related proteins that are involved in RNA processing—a surprising observation since extensive splicing machinery seems to be absent in noneukaryotes.

**What led you to study RNA or this aspect of RNA science?**

Our foray into the field of SR/SR-related proteins was a bit unconventional. In the past, our laboratory has generally focused on prion and prion-like domains. A few years ago, we identified a prion-like domain in a yeast SR kinase, Sky1, that mediated its recruitment to stress granules. That study catalyzed our initial interest in SR/SR-related proteins. Shortly thereafter, SARS-CoV-2 emerged and we formally proposed early on that the viral nucleocapsid protein (which contains an RS domain) would be involved in regulating stress granules in human cells during infection. At the same time, we were also developing a bioinformatic tool, LCD-Composer, that was designed to identify any type of low-complexity domain—including RS domains—with high specificity. We were aware that identifying SR/SR-related proteins has been a challenging task historically, and we realized we now had the perfect tool to search for new SR-related proteins.

While LCD-Composer was the launch pad for this study, in the larger context it was really the convergence of these three projects that led us to explore SR/SR-related proteins in greater depth. This has helped me realize that many threads often connect fields of study that may seem unrelated, and one of the joys of science is finding and following those threads!

**During the course of these experiments, were there any surprising results or particular difficulties that altered your thinking and subsequent focus?**

I expected to find at least a few new SR-related proteins, but I was surprised at just how many our method uncovered and how similar they were in terms of biological activity (based on existing experimental evidence) to the previously known SR/SR-related proteins. The functional domains of SR-related proteins shared across archaea, bacteria, and eukaryotes were also a bit surprising and fun to explore.

**If you were able to give one piece of advice to your younger self, what would that be?**

Lean into your curiosity as a primary motivator: it is a renewable resource, it encourages you to look around every corner and under every rock, and it usually operates in a positive-feedback loop. Continuous growth, learning, and skill acquisition/development are also natural by-products of following your curiosity.
