# Supplementary material for: Expansion and functional analysis of the SR-related protein family across the domains of life
Source: RNA. 2022 Oct;28(10):1298–314. doi: 10.1261/rna.079170.122 (PMC9479744; doi:10.1261/rna.079170.122)
Supplement: Supplemental Material [file supp_28_10_1298__DC1.html]

Expansion and Functional Analysis of the SR-Related Protein Family Across the Domains of Life — Expansion and functional analysis of the SR-related protein family across the domains of life — Supplemental Material 

# Expansion and functional analysis of the SR-related protein family across the domains of life

## Supplemental Material

- Supplemental\_Figures.zip
- Supplemental\_Legends.docx
- Supplemental\_Tables.zip
