# Supplementary figures and images for: Expansion and functional analysis of the SR-related protein family across the domains of life
Source: RNA. 2022 Oct;28(10):1298–314. doi: 10.1261/rna.079170.122 (PMC9479744; doi:10.1261/rna.079170.122)

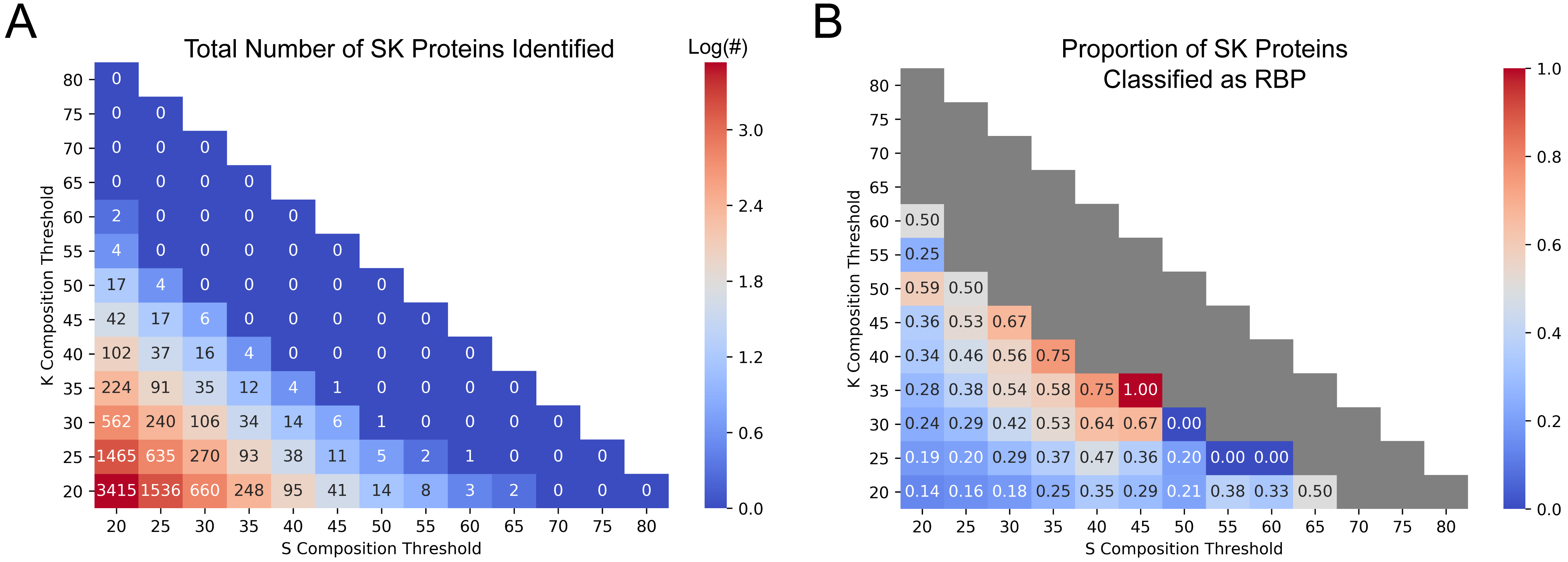

Supplement: Supplemental Material [file supp_079170.122_Supplemental_Figures.zip › Supplemental_Fig_S1.tif]

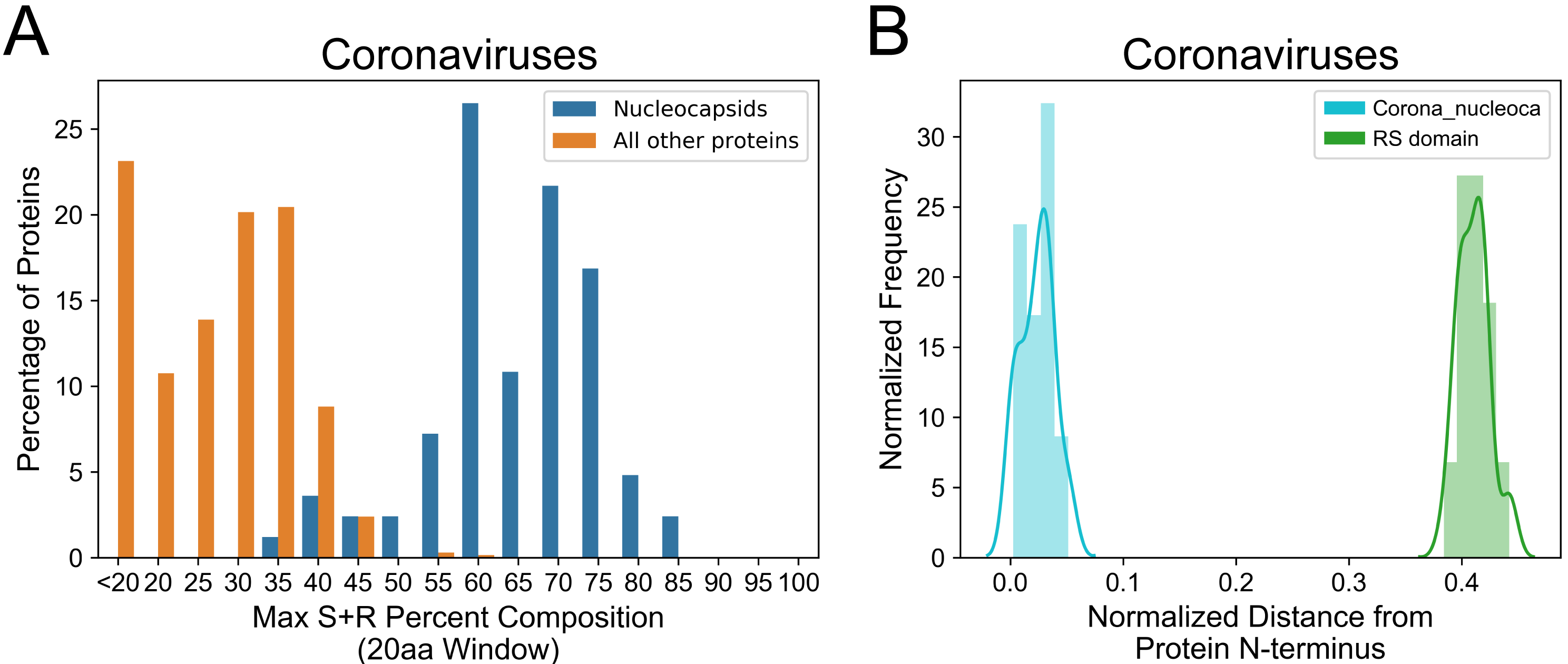

Supplement: Supplemental Material [file supp_079170.122_Supplemental_Figures.zip › Supplemental_Fig_S10.tif]

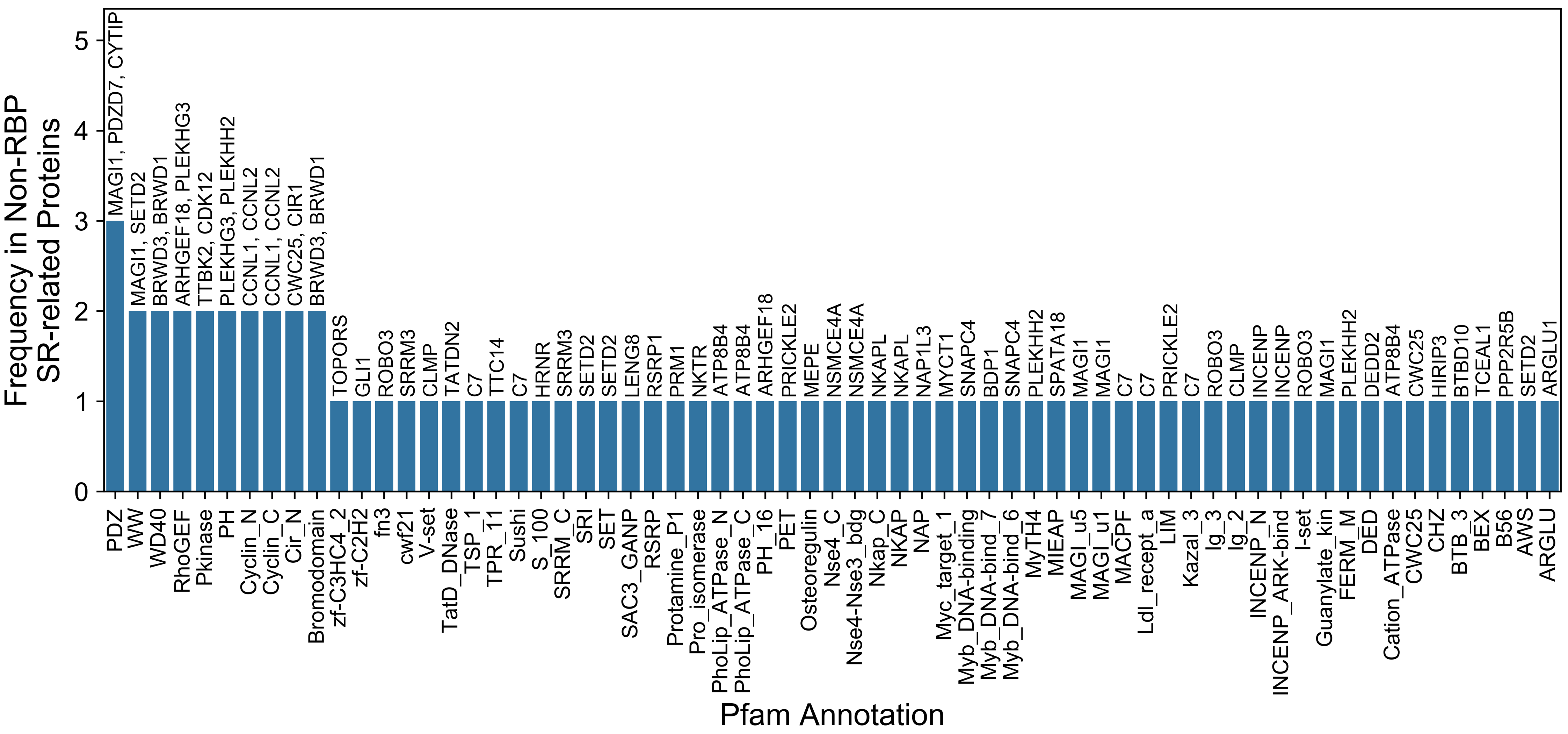

Supplement: Supplemental Material [file supp_079170.122_Supplemental_Figures.zip › Supplemental_Fig_S11.tif]

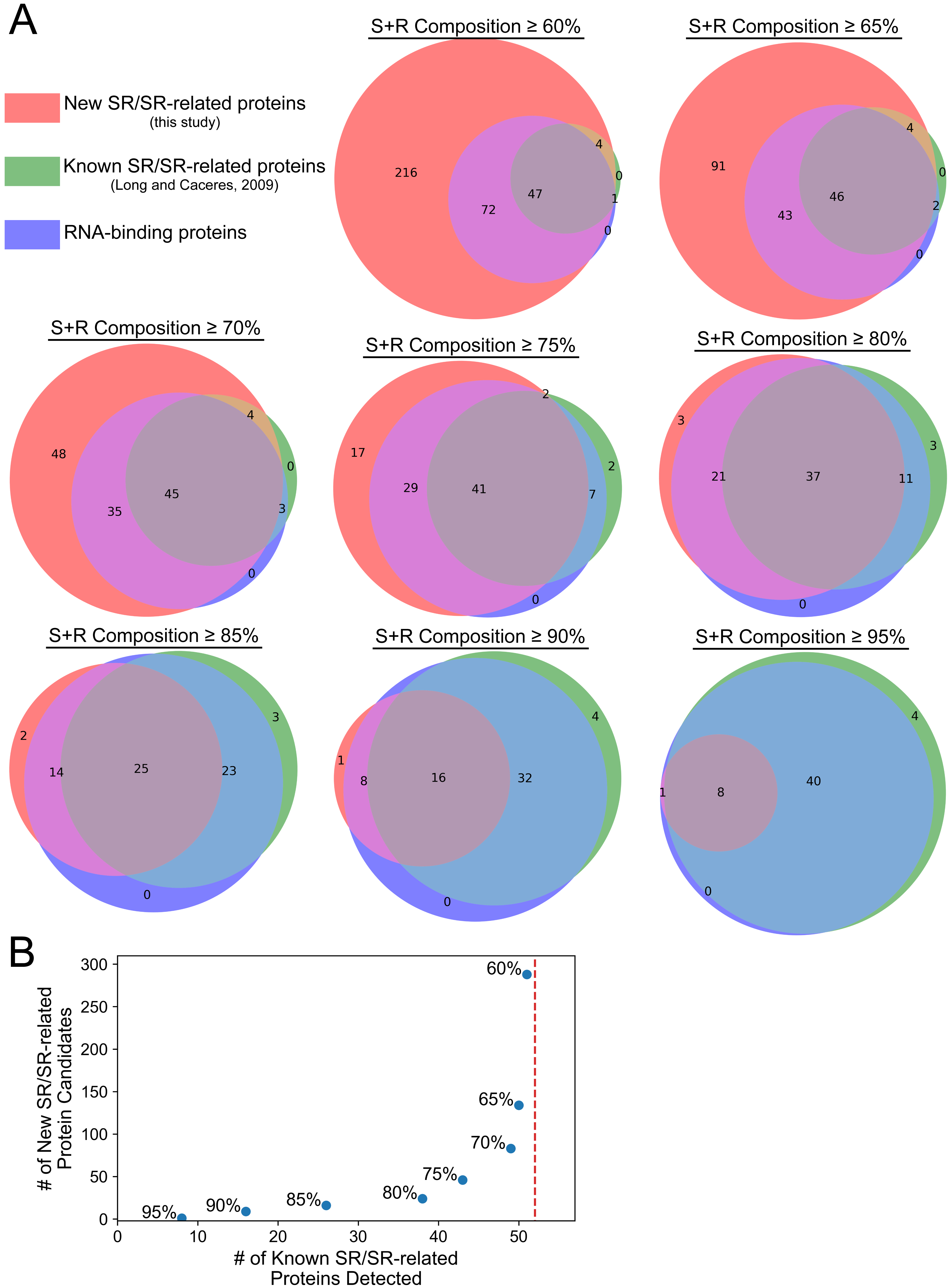

Supplement: Supplemental Material [file supp_079170.122_Supplemental_Figures.zip › Supplemental_Fig_S2.tif]

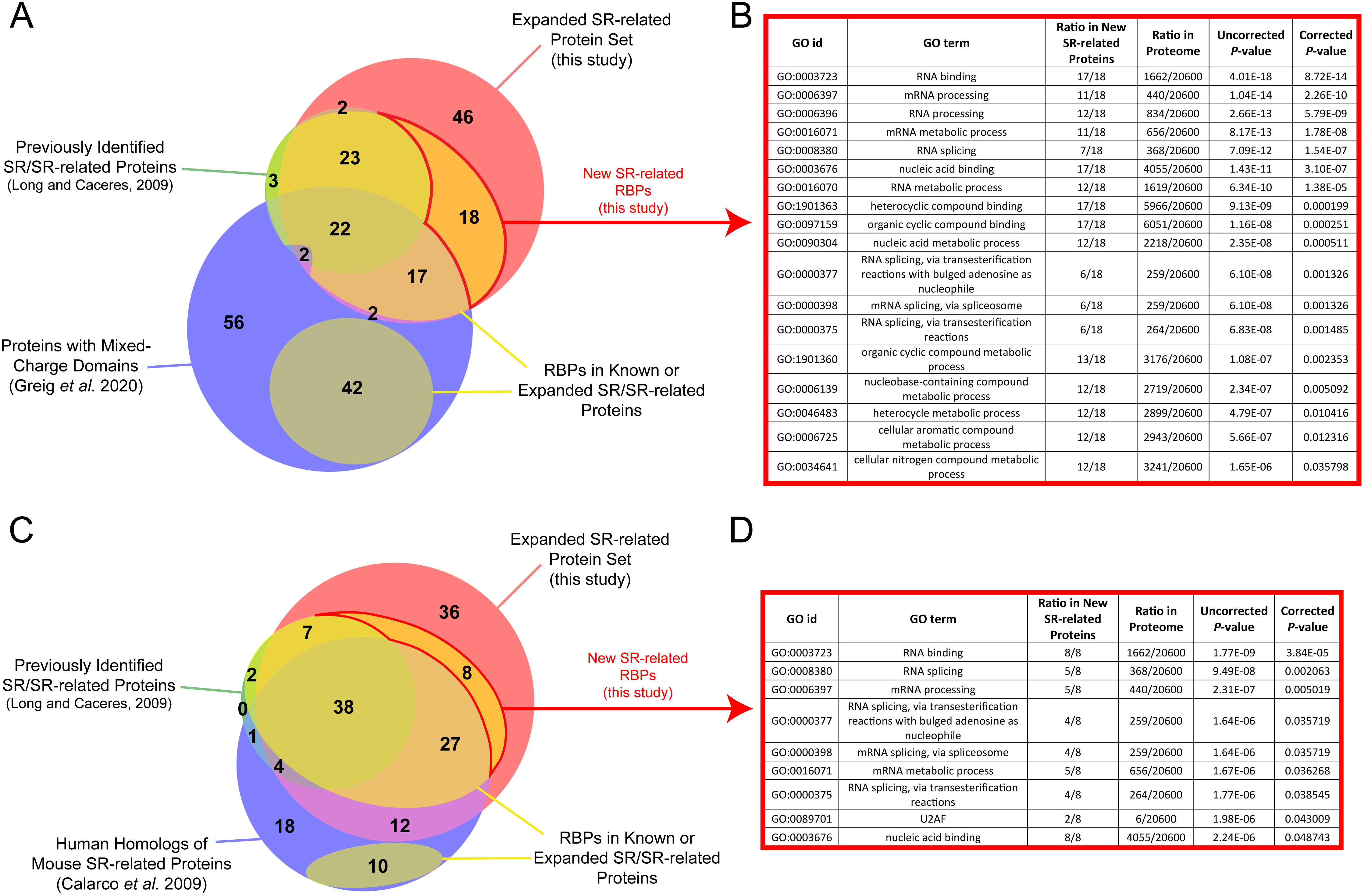

Supplement: Supplemental Material [file supp_079170.122_Supplemental_Figures.zip › Supplemental_Fig_S3.tif]

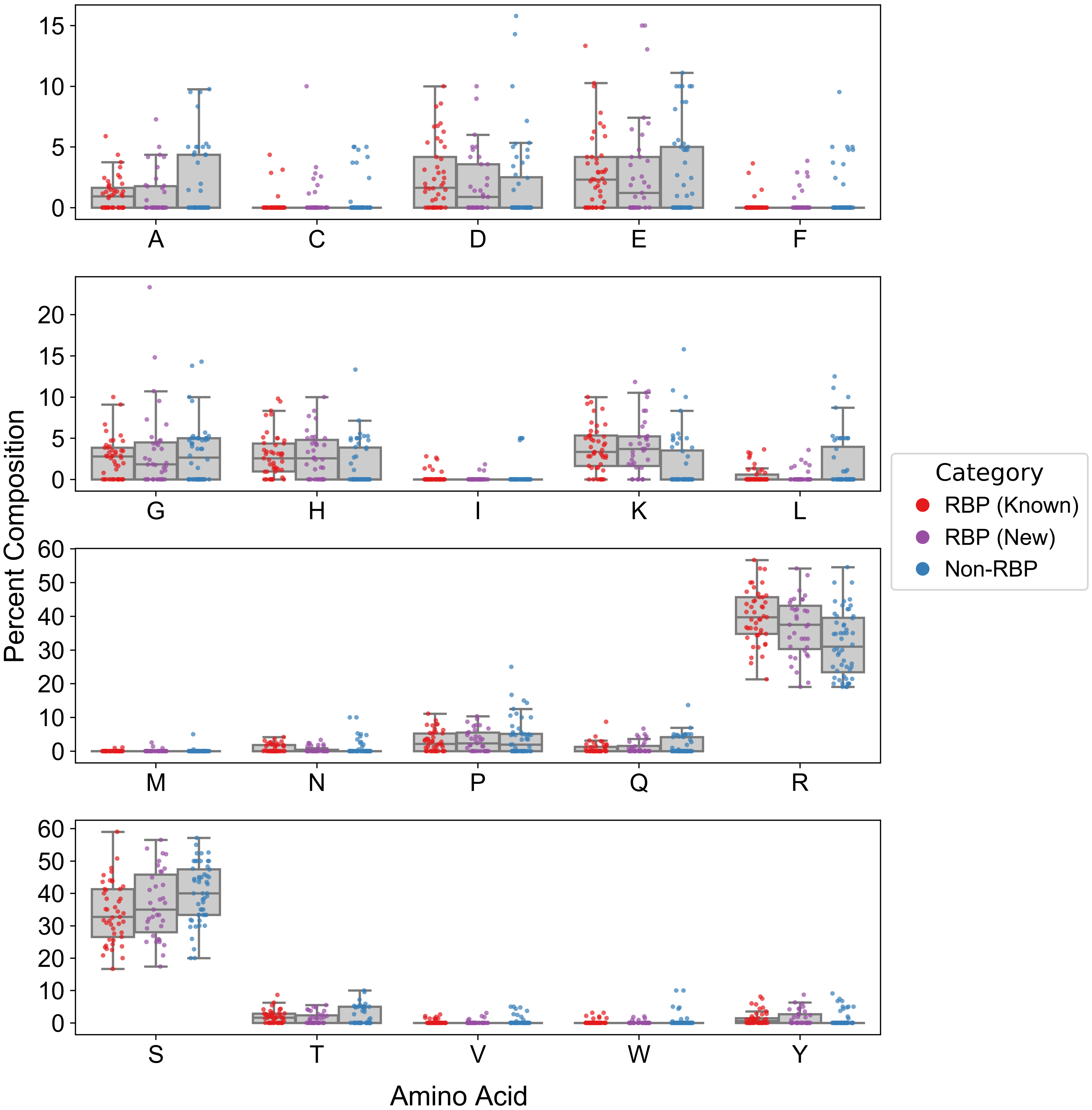

Supplement: Supplemental Material [file supp_079170.122_Supplemental_Figures.zip › Supplemental_Fig_S4.tif]

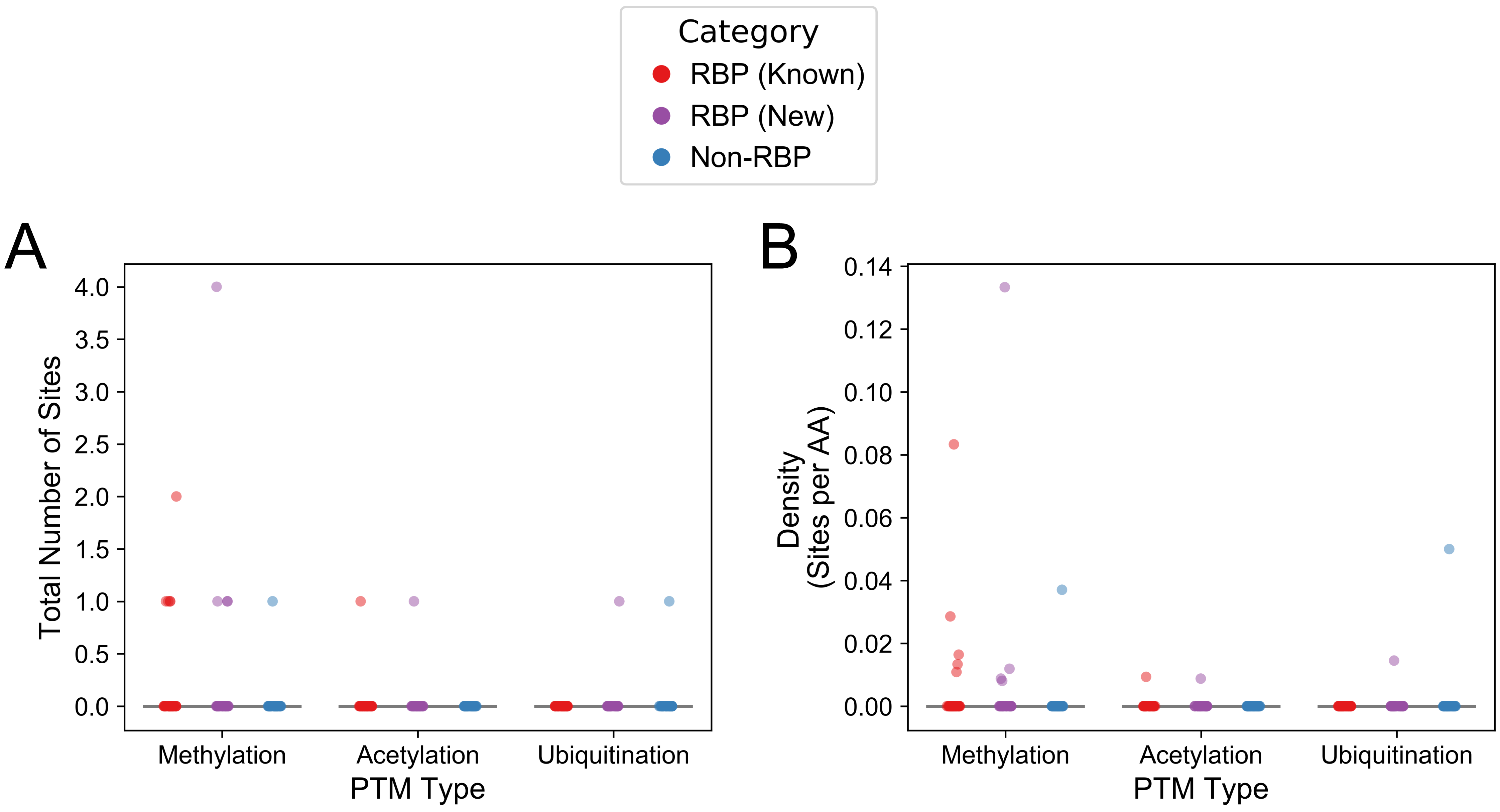

Supplement: Supplemental Material [file supp_079170.122_Supplemental_Figures.zip › Supplemental_Fig_S5.tif]

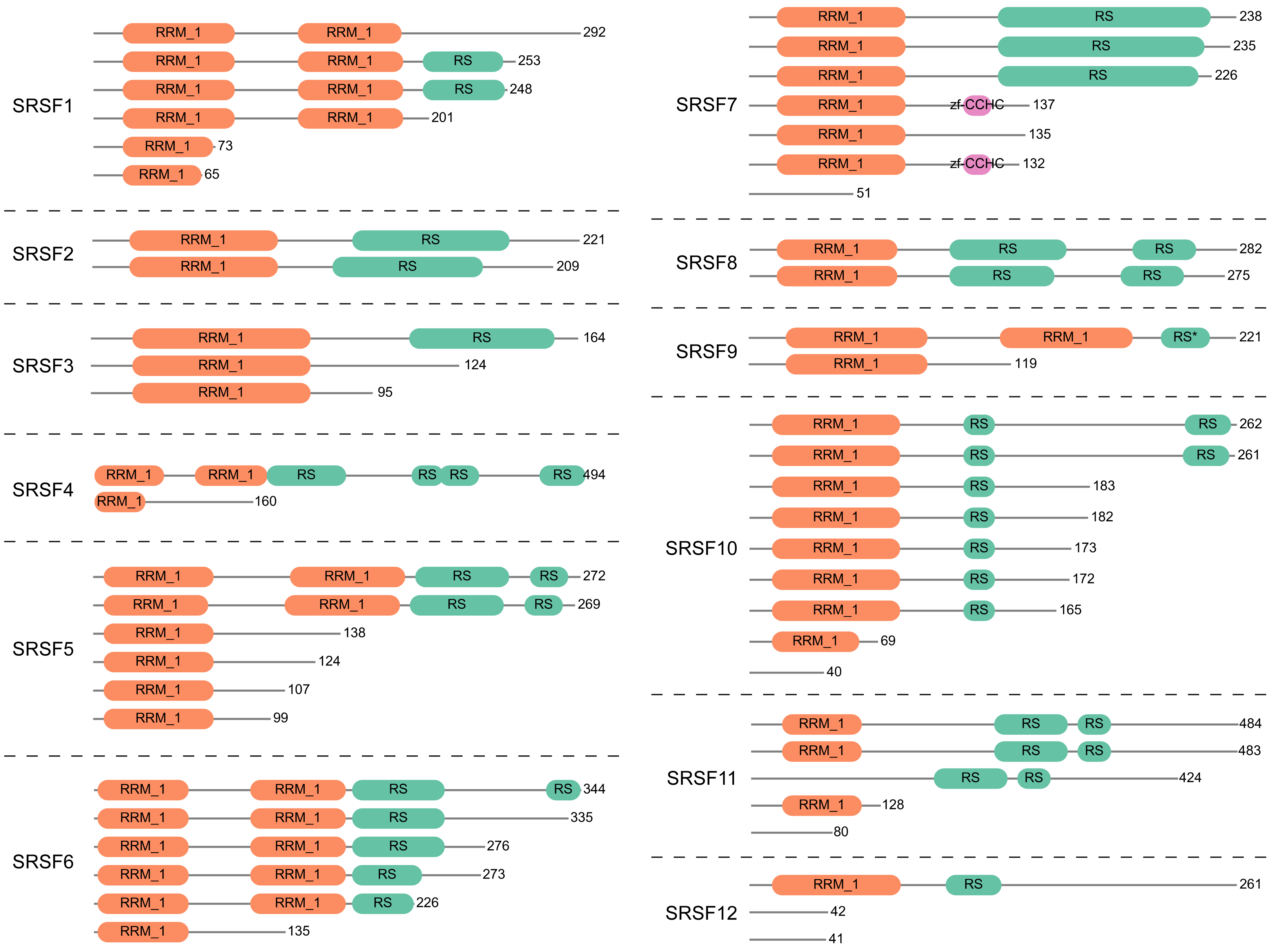

Supplement: Supplemental Material [file supp_079170.122_Supplemental_Figures.zip › Supplemental_Fig_S6.tif]

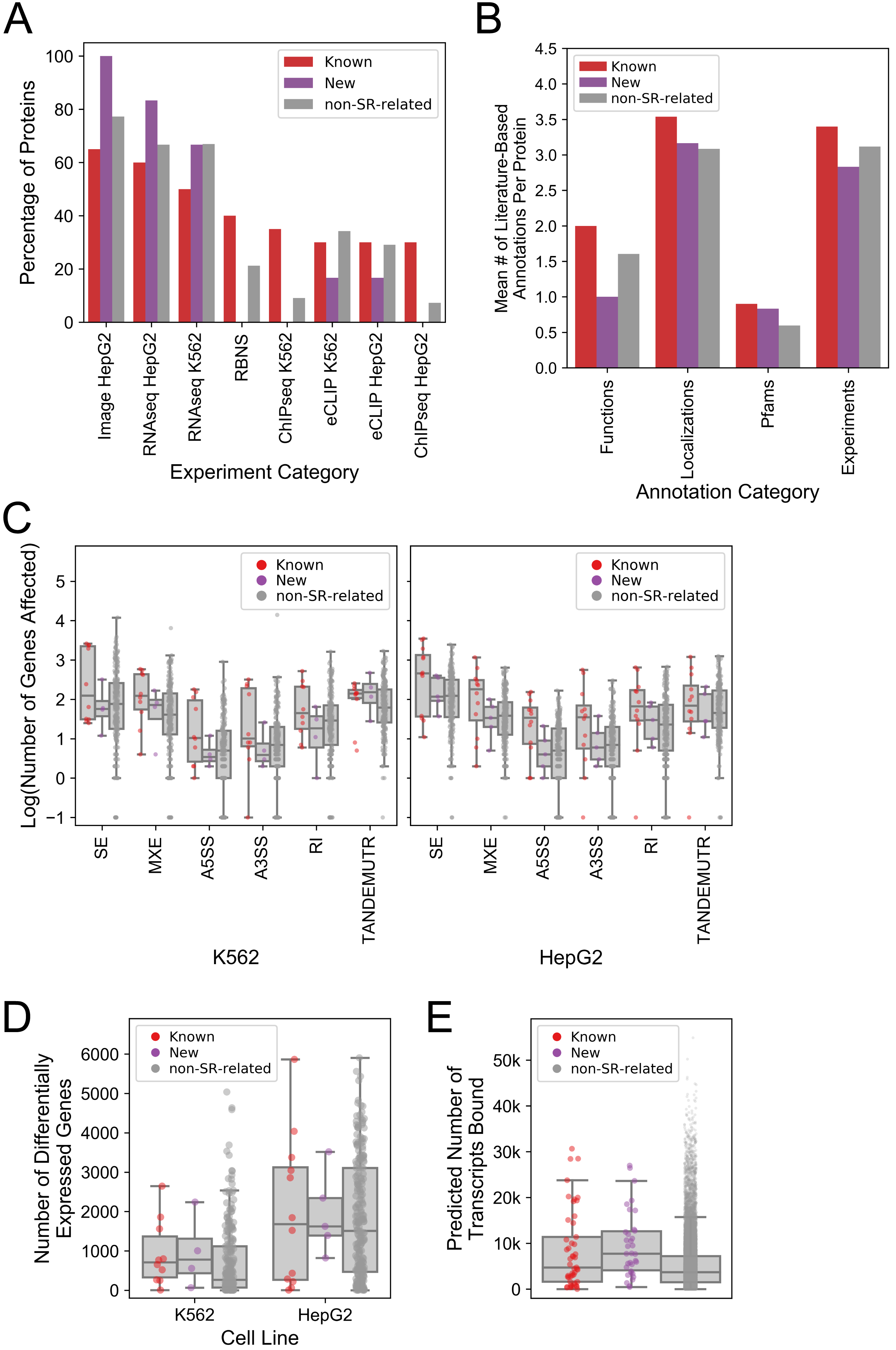

Supplement: Supplemental Material [file supp_079170.122_Supplemental_Figures.zip › Supplemental_Fig_S7.tif]

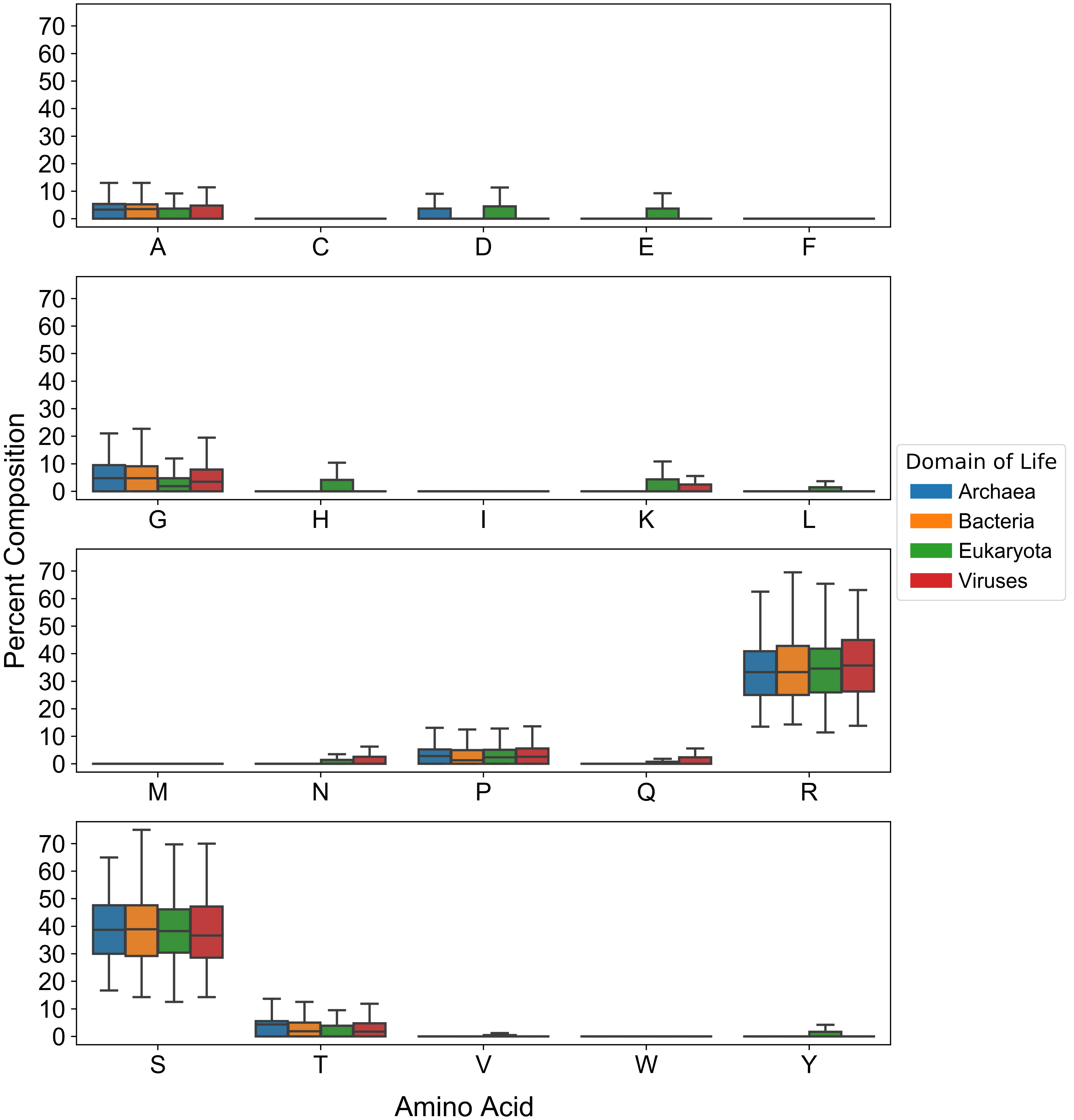

Supplement: Supplemental Material [file supp_079170.122_Supplemental_Figures.zip › Supplemental_Fig_S8.tif]

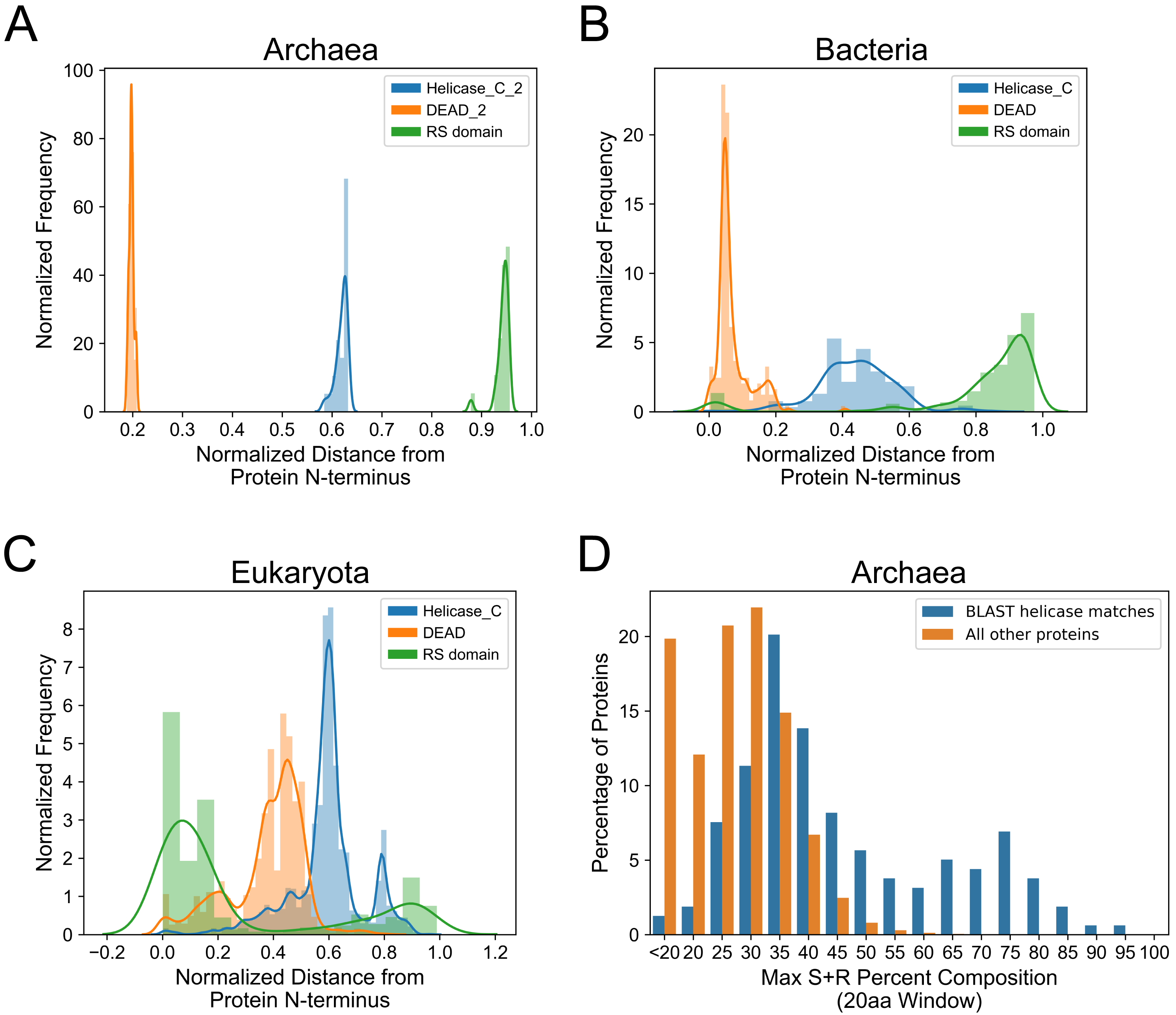

Supplement: Supplemental Material [file supp_079170.122_Supplemental_Figures.zip › Supplemental_Fig_S9.tif]
