# Supplementary material for: Expansion and functional analysis of the SR-related protein family across the domains of life
Source: RNA. 2022 Oct;28(10):1298–314. doi: 10.1261/rna.079170.122 (PMC9479744; doi:10.1261/rna.079170.122)
Supplement: Supplemental Material [file supp_079170.122_Supplemental_Legends.docx]

**Supplemental Fig. S1. Identification of KS domains in human proteins.** Iterative composition analyses were performed as indicated for Fig 1 using lysine instead of arginine as the positively charged residue. (A) Number of proteins identified for each K and S composition threshold criterium. (B) The proportion of identified proteins with KS domains that are classified as RBPs by Gerstberger *et al.* (Gerstberger et al. 2014), contain an RRM, or are directly annotated with the GO term “RNA binding”.

**Supplemental Fig. S2. Evaluation of alternative S+R composition thresholds for defining human SR/SR-related proteins.** (A) For all combined S+R composition thresholds from 60-95% (in 5% increments), identified proteins were pooled and evaluated for degree of overlap with the SR/SR-related proteins defined in (Long and Caceres 2009) and with known RBPs. For simplicity, RBPs not classified as known or new SR/SR-related proteins are omitted. Additionally, one protein is omitted from the 80% S+R and the 85% S+R diagrams due to geometric constraints when drawing the Venn diagrams: this protein is identified in this study and by Long and Caceres but is not classified as an RBP. (B) Scatter plot depicting the number of new vs. known SR/SR-related proteins for each combined S+R composition threshold (values adjacent to data points). The dotted red line indicates the total number of known SR/SR-related proteins. At combined S+R composition thresholds below 70%, there is a sharp increase in the number of new SR/SR-related proteins with only marginal gains in the number of known SR/SR-related proteins detected.

**Supplemental Fig. S3. Comparison of our SR/SR-related proteins to related, published sets of SR/SR-related proteins.** (A) Overlap between our identified SR/SR-related proteins, the defined SR/SR-related proteins from (Long and Caceres 2009), proteins with mixed-charge domains [including phosphorylated RS domains; (Greig et al. 2020)], and known RBPs. (B) GO term analysis for 18 SR-related RBPs not found in the mixed-charge dataset or Long and Caceres dataset. (C) Overlap between our identified SR/SR-related proteins, the defined SR/SR-related proteins from (Long and Caceres 2009), human homologs of mouse SR/SR-related proteins (Calarco et al. 2009), and known RBPs. (D) GO term analysis for 8 SR-related RBPs not found in the Calarco *et al.* dataset or Long and Caceres dataset.

**Supplemental Fig. S4. Full composition analysis of RS domains.** Boxplots depicting the percent composition of all 20 canonical amino acids within the RS domains of known, new, and non-RBP SR/SR-related proteins (as defined in the Fig 2 legend).

**Supplemental Fig. S5. Frequencies of non-phosphorylation PTMs in RS domains.** Total number of known methylation, acetylation, and ubiquitination sites (A) and site densities (B) within RS domains of known, new, and non-RBP SR/SR-related proteins (as defined in the Fig 2 legend).

**Supplemental Fig. S6. The RS domains of all human SRSF proteins are affected by alternative splicing and other forms of sequence variation.**

**Supplemental Fig. S7. Experimental coverage of known and new SR/SR-related proteins and their effects on splicing, gene expression, and transcript binding.** (A) Percentage of new and known SR/SR-related proteins, as well as non-SR-related proteins, characterized in each type of experiment in the K562 or HepG2 cell lines. (B) Mean number of annotations for each characterized protein across different annotation categories. (C) Number of alternative splicing events affected upon knockdown of each RBP in either K562 or HepG2 cells, classified by type of alternative splicing (x-axis; SE: skipped exon, MXE: mutually exclusive exons, A5SS: alternative 5′ splice sites, A3SS: alternative 3′ splice sites, RI: retained intron, and TANDEMUTR: tandem 3′UTR). (D) Number of differentially expressed genes observed upon knockdown of each RBP. (E) Predicted number of transcripts bound by each RBP. Panels A-D were derived from Van Nostrand *et al.* (2020), whereas panel E is derived from the RNAct database (Lang et al. 2019).

**Supplemental Fig. S8. Full composition analysis of RS domains.** Boxplots depicting the percent composition of all 20 canonical amino acids within the RS domains from archaea, bacteria, eukaryotes, and viruses.

**Supplemental Fig. S9. SR-related helicase proteins have consistent domain architectures and orthologs with high S+R content.** Protein domain architecture for the identified SR-related helicases in archaea (A), bacteria (B), and eukaryota (C). RS domains of SR-related helicases are found almost exclusively at the C-terminus in archaea and bacteria but could be found at either terminus in eukaryotes (with some containing separate RS domains at both termini). (D) Comparison of the maximum S+R percent composition with a 20-residue window achieved by archaeal SR-related helicase orthologs versus all other archaeal proteins.

**Supplemental Fig. S10. SR-related coronavirus nucleocapsid proteins have consistent domain architectures and orthologs with high S+R content.** (A) Comparison of the maximum S+R percent composition with a 20-residue window achieved by coronavirus nucleocapsid proteins versus all other coronavirus proteins. (B) Protein domain architecture for coronavirus nucleocapsid proteins. Note that, although coronavirus nucleocapsid proteins are only associated with 1 Pfam annotation (“Corona_nucleoca”), these proteins generally have 2 canonical, functionally important structured domains, with the 2^nd^ structured domain on the C-terminal side of the RS domain (Cascarina and Ross 2022).

**Supplemental Fig. S11. Pfam annotation frequencies among non-RBP SR-related proteins in humans.**

**Supplemental Table S1. RS domains and associated characteristics for human proteins.** Contains information on RS domain-containing proteins, RS domain boundaries, the composition thresholds with which the protein was identified, whether the protein is classified as an RBP, SR/RS dipeptide counts within the RS domain, and PTMs mapping to the RS domains.

**Supplemental Table S2. GO term analysis for the previously known SR/SR-related proteins.** The set of 52 proteins defined as SR/SR-related proteins [(Long and Caceres 2009); see Fig 1C] were evaluated for GO term enrichment.

**Supplemental Table S3. GO term analysis for human proteins with comparable S-rich-only domains.**

**Supplemental Table S4. GO term analysis for human proteins with comparable R-rich-only domains.**

**Supplemental Table S5. Proteins identified at each S+R combined composition threshold.** The numbers of proteins, along with the associated protein identifiers, for each Venn diagram section in Supplemental Fig. S2A are indicated for each S+R combined composition threshold.

**Supplemental Table S6. Comparison of GO term enrichment for SR-related proteins identified with a 60%, 65%, and 70% S+R combined composition threshold.**

**Supplemental Table S7. Mapping of RS domains across all human protein isoforms.** Contains RS domain boundaries for all human protein isoforms, as well as binary indicators (0 or 1) of whether all protein isoforms contain an RS domain and whether all extant RS domains perfectly align for each isoform.

**Supplemental Table S8. RS domains and Pfam annotations among SR-related archaeal proteins.**

**Supplemental Table S9. RS domains and Pfam annotations among SR-related bacterial proteins.**

**Supplemental Table S10. RS domains and Pfam annotations among SR/SR-related eukaryotic proteins.**

**Supplemental Table S11. RS domains and Pfam annotations among SR-related viral proteins.**

**Supplemental Table S12. Statistical comparison of top 10 Pfam annotations among SR/SR-related proteins relative to S-rich-only or R-rich-only proteins.**

**Supplemental Table S13. Extended composition scoring for archaeal proteins.** All archaeal proteins were scored based on the maximum S+R percent composition within a 20-, 30-, 40-, and 50-residue window for each protein. Proteins lacking a window with at least 10% S and at least 10% R for all of the aforementioned window sizes were omitted from the table to reduce file sizes. For all remaining proteins, a score of -1 was assigned if the protein did not contain at least one window with a minimum of 10% S and 10% R.

**Supplemental Table S14. Extended composition scoring for bacterial proteins.** All bacterial proteins were scored as indicated for Supplemental Table S13, except that proteins lacking a window at least 20% S and at least 20% R for all of the indicated window sizes were omitted from the table due to file size constraints.

**Supplemental Table S15. Extended composition scoring for eukaryotic proteins.** All eukaryotic proteins were scored as indicated for Supplemental Table S14.

**Supplemental Table S16. Extended composition scoring for viral proteins.** All viral proteins were scored as indicated for Supplemental Table S13.

**Supplemental Table S17. GO term enrichment results for SR-related non-RBPs.** GO term analysis was performed on all SR-related non-RBPs identified in this study (see Fig 1C).

**Supplemental Table S18. GO terms directly assigned to the human NKAP protein.**
